# Modeling the Insulin-Like Growth Factor System in Articular Cartilage

**DOI:** 10.1371/journal.pone.0066870

**Published:** 2013-06-26

**Authors:** Lihai Zhang, David W. Smith, Bruce S. Gardiner, Alan J. Grodzinsky

**Affiliations:** 1 Department of Infrastructure Engineering, The University of Melbourne, Victoria, Australia; 2 Faculty of Engineering, Computing and Mathematics, The University of Western Australia, Western Australia, Australia; 3 Center for Biomedical Engineering, Department of Electrical Engineering and Computer Science, Department of Mechanical Engineering, Massachusetts Institute of Technology, Cambridge, Massachusetts, United States of America; Rice University, United States of America

## Abstract

IGF signaling is involved in cell proliferation, differentiation and apoptosis in a wide range of tissues, both normal and diseased, and so IGF-IR has been the focus of intense interest as a promising drug target. In this computational study on cartilage, we focus on two questions: (i) what are the key factors influencing IGF-IR complex formation, and (ii) how might cells regulate IGF-IR complex formation? We develop a reaction-diffusion computational model of the IGF system involving twenty three parameters. A series of parametric and sensitivity studies are used to identify the key factors influencing IGF signaling. From the model we predict the free IGF and IGF-IR complex concentrations throughout the tissue. We estimate the degradation half-lives of free IGF-I and IGFBPs in normal cartilage to be 20 and 100 mins respectively, and conclude that regulation of the IGF half-life, either directly or indirectly via extracellular matrix IGF-BP protease concentrations, are two critical factors governing the IGF-IR complex formation in the cartilage. Further we find that cellular regulation of IGF-II production, the IGF-IIR concentration and its clearance rate, all significantly influence IGF signaling. It is likely that negative feedback processes via regulation of these factors tune IGF signaling within a tissue, which may help explain the recent failures of single target drug therapies aimed at modifying IGF signaling.

## Introduction

**Figure 1:**
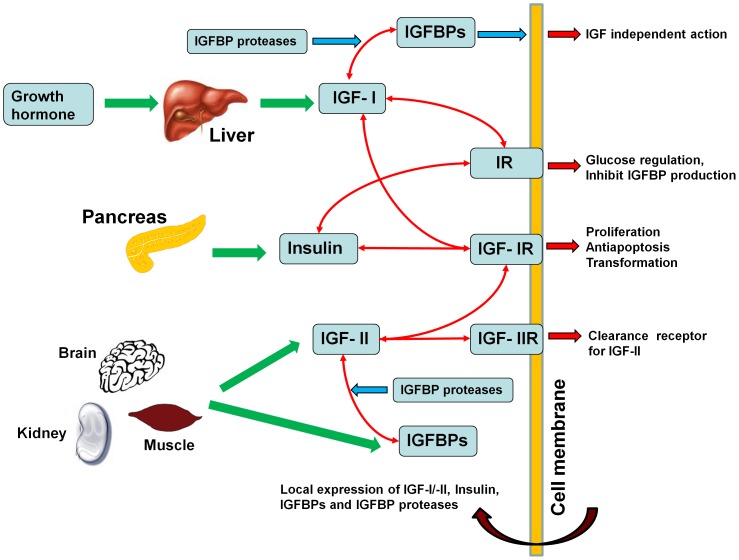
Schematic diagram of the IGF system.

The insulin-like growth factor system is comprised of two insulin-like growth factors (*i.e.* IGF-I and –II), type I and II IGF receptors (*i.e.* IGF-IR and IGF-IIR), insulin receptor (IR), a family of IGF binding proteins (here we focus on IGFBP1 through to IGFBP6) and IGFBP-degrading proteases [1] (see Figure 1 for schematic). Growth hormone regulates the IGF-I production by the liver, which is the source of the majority of IGF-I found in plasma [2]. On the other hand, IGF-II and IGFBPs found in the serum are most likely sourced from a variety of tissues (*e.g.* liver, muscle, brain, kidney being the principal sources) [3], [4].

IGF signalling through the type I IGF receptor (IGF-IR) is involved in cell proliferation, differentiation, apoptosis and general anabolic cell processes (including the production of extra cellular matrix) [5]. An absence of IGF leads to growth hormone resistant growth failure, which may be treated using the synthetic IGF mecasermin [6]. A low level of IGF-I has also been shown to be associated with insulin-dependent diabetes in children and cardiovascular disease in adults [7].

Excessive levels of IGFs in the circulation are linked with an increased risk of cancer [1], [8], [9], [10], and there is some compelling evidence that the IGF/IGF-IR system plays a major role in some types of human neoplasm [11], [12]. Intervening in the IGF signaling system has been identified as an attractive strategy for the treatment of certain human cancers [13]. For example, the reduction in IGF-IR activation by the binding of specific antibodies leads to apoptosis of cancer cells [14], [15]. A recent study using the monoclonal antibody ‘Figitumumab’, supported the potential therapeutic efficacy of anti-IGF-IR strategies for the treatment of patients with Ewing's sarcoma [16]. However several drug companies have recently stopped development of drugs designed to block IGF-R signaling, expressing frustration over the ineffectiveness of drugs that have been developed, blaming the biological complexity of the IGF system [17]. Based on the hard won (negative) findings, it is now clearly apparent that a ‘systems approach’ is needed to understand why a single drug target may be ineffective for managing IGF-IR signaling. Indeed, it points to the fact that several drugs acting together may be required to effectively block a signaling pathway. From a sensitivity analysis for our model, we find that it is likely that negative feedback processes act to neutralize the effect of attempting to block a single target.

It is expected that treatment of patients with a variety of disease processes in all tissues of the body may be enhanced when there is an improved understanding of the processes that regulate the cell's exposure to IGF within a tissue from a circulating source of IGF. To contribute towards this goal, this paper is focused developing a systems model to estimate the free and total IGF concentrations within a single tissue – articular cartilage. Cartilage was chosen primarily because we are aware of appropriate data to enable calibration of the model.

IGF-I and IGF-II (and insulin in high concentrations) bind to the IGF-IR receptor, leading to activation of a receptor tyrosine kinase and subsequent downstream signaling via the AKT pathway. The strength of activation of the signaling (for a fixed receptor concentration) depends on the fraction of overall receptors that have formed a complex with their ligands. However the downstream pathway activation need not be proportional to the receptor occupancy. For example, previous studies on cartilage have shown there is a certain threshold of IGF-I/ IGF-IR concentration that needs to be exceeded before protein synthesis is activated [18], [19]. In this paper IGF-IR complex formation is included, but no downstream signaling processes are modeled, so the IGF-IR complex concentration is adopted here as the primary biological marker of functional activity due to IGFs in the tissue.

Although IGF-II is widely argued to play an important role in embryonic and foetal life [1], [20], recent studies indicate that IGF-II is also important in adults for muscle, brain and other tissues by signaling through the receptor IGF-IR [4], [21]. While many tissues produce IGF-II, most tissues produce little or no IGF-I, with the majority of IGF-I in tissues originating from production by the liver [22]. Only IGF-II binds to the IGF-IIR receptor [23]. Formation of IGF-IIR complexes usually has no known downstream signaling consequences, although it has been reported that binding of IGF-II to IGF-IIR may provide a possible mechanism for the regulation of cardiomyocyte apoptosis [24]. Instead it is thought that the primary role of the IGF-II-IGF-IIR complex is the regulation of the IGF-II concentration in the tissue, *i.e* via sequestration and removal of the IGF-II-IGF-IIR complex through lysosomal degradation [25]. In other words, IGF-IIR is postulated to be a ‘clearance receptor’.

One conceivable mechanism for regulating the IGF-IR receptor complex concentration in the tissue (and so the IGF signaling pathway) is to regulate the ratio of IGF-I and –II in the tissue, as IGF-I and –II ligands competitively bind to IGF-IR. Note the IGF-II concentration in human plasma is typically three-fold higher than that of IGF-I [26], and the ratio of IGF-II/IGF-I may reach over 300 in a tumor [27]. The functional significance of these observations of IGF-II/IGF-I is yet to be fully appreciated.

A second possible mechanism for regulating the bioavailability of the two IGFs to IGF-IR is to adjust the type of IGFBPs within the tissue, *e.g.* by regulating the production of IGFBPs or the removal of IGFBPs. Among the ten current known IGFBPs, at least six of them (*i.e.* IGFBPs 1–6) bind IGFs with high affinity [28], [29], [30], [31]. While the full range of functional roles of the binding proteins remains to be clarified, some of their actions are known. First, IGFBPs can function as IGF carriers, protecting the IGFs from degradation while they are being transported through tissues [3], [32]. It is well known that binding proteins can also act as stores of IGFs within the tissue, which helps to smooth any fluctuations in IGF production or transport over time [3].

It has been demonstrated theoretically, using a reactive-diffusion transport model, that reversible binding between IGFs and diffusible IGFBPs can significantly increase the uptake rate of free IGF into a tissue) [33]. Most importantly, targeted degradation of IGF binding proteins can lead to substantial increases in the free IGF concentration in the tissue, compared to the concentration in the plasma, with the rate of degradation of the binding proteins controlling the free IGF concentration in the tissue [34]. That is, tissue can potentially tune their exposure to IGF by modifying the rate of degradation of the IGF binding partner.

Different IGFBP proteases may selectively target IGFBPs for degradation, potentially giving fine control over the total IGF concentration in the tissue and the ratio of IGF-I/IGF-II. For example, serine protease is reported to be mainly responsible for cleavage of IGFBP5 [35], whilst metalloproteinase ADAM 12-S primarily degrades IGFBP3 and IGFBP5 but not IGFBP1, −2, −4 and −6 [36]. In addition, matrix metalloproteinases (MMPs) are capable of increasing bioavailability of IGF-I by degrading IGFBP 1, −3, and −5 [37]. IGFBP6 is an O-linked glycoprotein. It is known that O-glycosylation inhibits human IGFBP6 degradation by chymotryspin and tryspin [38]. In addition, O-glycosylation also helps maintain IGFBP6 in soluble form by inhibiting its binding to glycosaminoglycans and cell membranes [38]. These targeted mechanisms provide tissue with the means to adjust their free IGF concentration. That is, cells in tissues can ‘tune’ their IGF exposure, effectively independently to the plasma concentration, to suit the tissue's particular needs. It is expected that these tuning processes would contribute to the maintenance of tissue homeostasis.

IGFBPs are also capable of blocking IGFs access to IGF receptors (*e.g.* IGF-IR) through sequestration. IGFs have a 2–50 fold greater affinity for IGFBPs than that of the IGF-IR receptor itself [1], [39]. It has been theoretically demonstrated that extracellular matrix (ECM) fixed IGFBPs within the tissue have no influence on the *steady-state* free IGF-I and –II concentrations in the tissue if the half-lives of these ECM fixed IGFBPs are prolonged by ECM proteins [33]. IGF-independent cellular actions of the IGFBPs have also been reported [3], [34].

Among six IGFBPs (*i.e.* IGFBP1-6), IGFBP1-5 have approximately similar affinities for IGF-I and –II, but IGFBP6 has a 20–100 fold higher affinity for IGF-II than for IGF-I [40], [41], [42]. Because of the similar affinities, as a good approximation for many purposes, one may simply sum the concentrations of IGFBP1–5, and treat this as one functional group of BPs, and treat IGFBP6 as a second functional group. In our previous study [43], we have theoretically demonstrated that Bhakt *et al*'s experimental results for equilibrium competitive binding [44] can be successfully reproduced using a reversible Langmuir sorption isotherm involving these two ‘functional groupings’ of IGFBPs. The effect of this competitive binding on ligand and complex formation will be included in this study.

A third possible mechanism to regulate the IGF-IR receptor complex concentration in the tissue is to regulate the IGF-IR receptor density at the cell surface. Given a constant IGF concentration, as the receptor density increases, so the total number of IGF-IR receptor complexes will clearly increase. There is also the possibility that cells may spatially vary their expression of cell surface receptors throughout the tissue, which adds another layer of complexity. This is an important area and will later on be investigated in a parametric study.

Receptor behavior is complex. IGF-IR has significantly higher binding preference for IGF-I and –II compared to insulin, whereas IGF-IIR only preferentially binds IGF-II [23]. In comparison to IGFBPs 1–6, IGFBP-7 lacks the important ternary structure required for binding IGFs with high affinity, but has the capability of binding to insulin and subsequently inhibit insulin binding to the insulin receptor (IR) [45]. Although IGFBP-7 has been identified in human biological fluid, its concentration is too small to detect in human cartilage [46], and so is not explicitly considered in our model. The insulin receptor primarily regulates cell metabolic functions [47]. Both IGF-IR and insulin receptors are usually tyrosine kinase homodimers, but IGF-IR-insulin heterodimers may form [5]. Hybrid receptors (IGF-IR/IR) formed by IGF-IR and IR bind to IGF-I with at least 50-folder higher affinity than insulin irrespective of the splice variant [48]. Homo- and hetero-dimerisation of receptors is not considered here.

While much is known about the individual components making up the IGF system, it still remains unclear how these components act together as an integrated system within a tissue. Indeed, it is likely that a ‘systems approach’ is required for the development of more efficacious drug therapies. Our previous studies of cartilage have been particularly focussed on the IGF-I mediated cartilage ECM biosynthesis via IGF-IR [18], [19], [33], [43], [49], [50], [51], [52], [53]. In this study, to achieve a system level of understanding of how tissues regulate their exposure to growth factors and so maintain normal tissue homeostasis and biological functions, we have developed a computational model of IGF system in cartilage involving IGF-I, IGF-II, insulin, IGF-IR, IGF-IIR and IR. Our aim is to identify the critical model variables for potentially controlling IGF signaling homeostasis based on a sensitivity analysis for the system. It is expected that the cartilage model developed here could be generalized further and applied to a range of different tissues in health and disease.

## Methods

**Figure 2:**
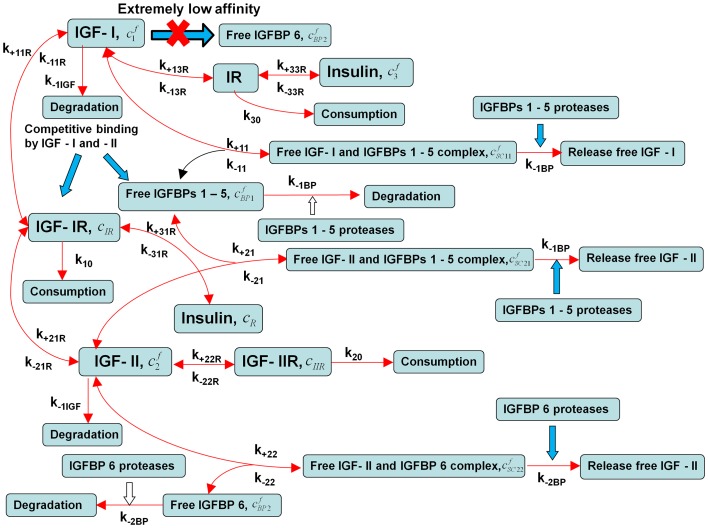
Schematic diagram shows the scope of this study.

The general outline of the IGF system is illustrated in Figure 1, while the specific IGF system model considered in this paper is illustrated in Figure 2. As shown in Figure 2, IGF-I and –II exert their biological actions via competitively binding to IGF-IR, whereas IGF-IIR mainly functions as an IGF-II ‘decoy’ receptor, which is cleared by lysosomal degradation. To help demonstrate the fundamental behaviours exhibited by the IGF system within a complex tissue like cartilage, a simplification of the real system is necessary. Here it is assumed that

- The bioavailability of two IGFs is regulated by two functional groups of IGFBPs [43], that is, one group of binding proteins has similar binding affinity to both IGF-I and –II (*i.e.* IGFBP1-5), whereas the second group has only binding preference for IGF-II (*i.e.* IGFBP6).
- Zero initial conditions are assumed within the cartilage for all components except cells (specifically chondrocytes) which are assumed to be uniformly distributed throughout the tissue, however, it is noted that the steady-state solutions reported here are independent of the initial conditions.
- Given that ECM bound IGFBPs have little influence on steady-state IGF concentration [33] and the quantities of IGFBP produced by human cartilage are relatively small in comparison to the amount supplied from the circulation [54], ECM fixed IGFBPs and the local expression of IGFBPs are not explicitly considered in this study.

Referring to Figure 2 and using the law of mass action [55], [56], [57], we obtained the following system of partial differential equations describing the co-diffusion of the two IGFs, insulin and the IGFBPs from synovial fluid into the cartilage and interacting with IGF-IR, IGF-IIR and IR within the tissue, namely.

### Free IGF-I/-II and insulin



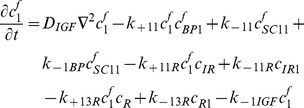
(1)

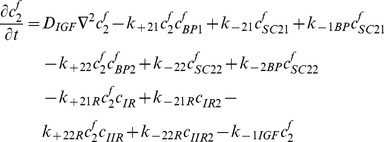
(2)

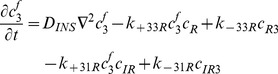
(3)Where 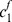 =  concentration of free IGF-I, 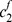 =  concentration of free IGF-II, 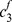 =  concentration of free Insulin, 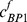  =  concentration of the first functional group of free IGFBPs (*i.e.* IGFBPs 1–5), 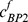  =  concentration of the second functional group of free IGFBPs (*i.e.* IGFBP-6), 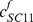  =  concentration of free IGF-I and IGFBPs 1–5 complex, 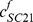  =  concentration of free IGF-II and IGFBPs 1–5 complex, 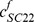  =  concentration of free IGF-II and IGFBP-6 complex, 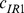  =  concentration of IGF-IR and IGF-I complex, 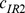  =  concentration of IGF-IR and IGF-II complex, 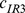  =  concentration of IGF-IR and Insulin complex, 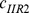  =  concentration of IGF-1IR and IGF-II complex, 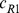  =  concentration of IR and IGF-I complex, 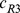  =  concentration of IR and Insulin complex, 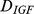  =  diffusion coefficient of free IGFs in tissue, 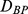  =  diffusion coefficient of free IGFBP in tissue, and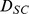  =  diffusion coefficient of free complex in tissue.

### Free two functional groups of IGFBP and their complexes



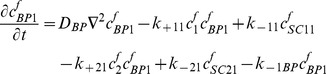
(4)

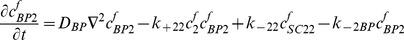
(5)

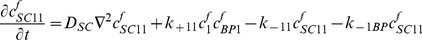
(6)

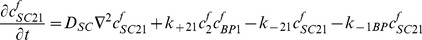
(7)

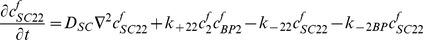
(8)


Note subscript ‘SC’ refers to the so-called ‘small binary complex’ formed between IGF and IGFBPs [58].

### IGFs and their receptors



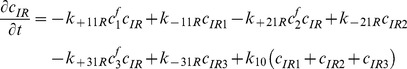
(9)

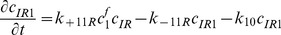
(10)

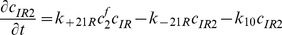
(11)

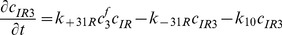
(12)

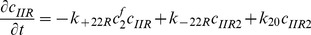
(13)

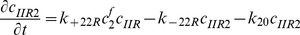
(14)

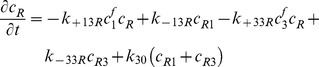
(15)

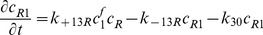
(16)

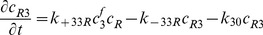
(17)Where 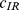  =  concentration of type I receptors (*i.e.* IGF-IR), 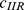  =  concentration of type II receptors (*i.e.* IGF-IIR), and 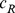  =  concentration of Insulin receptors (*i.e.* IR).

By adding Equations (9)–(12), (13)–(14), and (15)–(17) respectively, we obtain
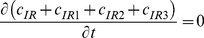
(18)

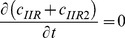
(19)

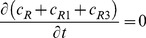
(20)Thus, 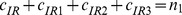, 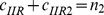 and 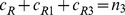, where 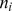 are constants which can be obtained from the initial condition, that is



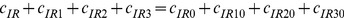
(21)

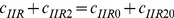
(22)

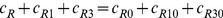
(23)where



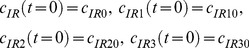
(24)




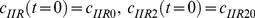
(25)

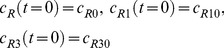
(26)Substituting equations (21)–(23) into equations (1)–(8) and (9)–(17) respectively, and by letting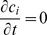, we obtain the following set of steady-state governing equations.

### Free IGF-I/-II and insulin



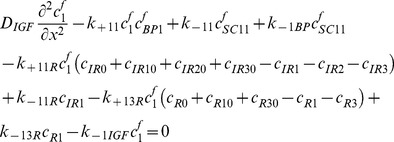
(27)

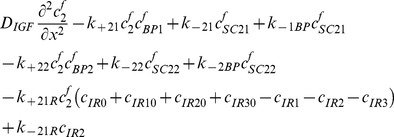
(28)

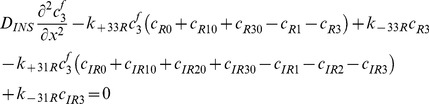
(29)


### Two functional groups of free IGFBP and their complexes



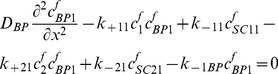
(30)

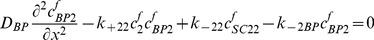
(31)

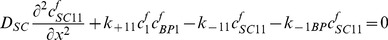
(32)

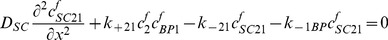
(33)

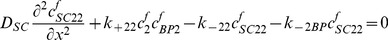
(34)


### IGF and their receptors



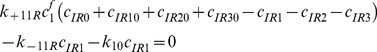
(35)

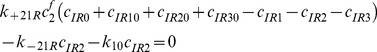
(36)

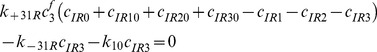
(37)

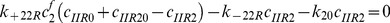
(38)

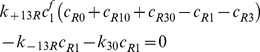
(39)

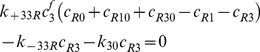
(40)


### Boundary conditions

At the cartilage surface (*i.e. x* = 0) it is assumed that IGF-I and –II are in a reversible equilibrium with their binding partners (*i.e.* IGFBPs) in synovial fluid. That is:
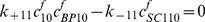
(41)

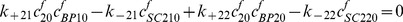
(42)

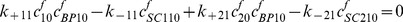
(43)

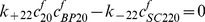
(44)

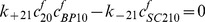
(45)where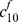, 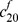, 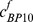, 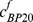, 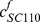,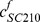 and 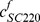 are concentrations of IGF-I and –II, two functional group IGFBPs and their complexes in synovial fluid respectively.

At the cartilage surface (*i.e. x* = 0) we assume that the concentration of IGF is continuous between the synovial fluid and the cartilage *i.e.* 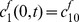
and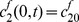. Due to the relatively large molecular size of IGFBPs and the small complex in relation to the pore openings at the surface of the cartilage, we treat IGFBPs differently to IGF. Specifically, the solute flux from fluid phase (*i.e.* synovial fluid) to the surface of the porous tissue (*i.e.* cartilage) can be characterized by a fluid phase mass transfer coefficient [59]. That is, here we assume the following mass flux boundary conditions to describe the relatively large molecules (*i.e.* IGFBPs and the small complexes) from the synovial fluid into the cartilage tissue:
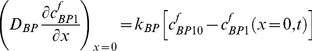
(46)

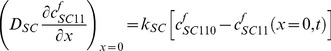
(47)

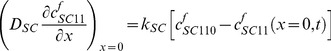
(48)

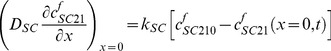
(49)

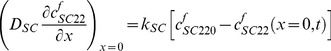
(50)where 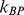 and 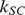 are mass transfer coefficients for IGFBP and small complex respectively. The mass transfer coefficient controls the transport of free IGFBP and the small complex between the synovial fluid and cartilage (porous) tissue.

At the bottom layer of the cartilage (i.e. *x* = 1.5 mm) (which is also the surface of subchondral bone), we assume the flux of all components equals zero (i.e. insulation boundary condition).

In this study, we specifically focus on two questions. First, what are the key factors (parameters) that govern the IGF-IR complex concentration within cartilage tissue? Second, how might cells regulate their IGF-IR complex concentration by the exposure to the two IGFs and insulin? To achieve these two objectives, we first calibrate the computational model by using experimental findings for the IGF system within the body and in articular cartilage. More specifically, the steady-state governing equations (27)–(40) were solved numerically using the commercial finite element software COMSOL stationary nonlinear solver [60] with the aim of obtaining a set of model parameters that could reproduce the observed experimental behavior in cartilage. Once calibrated, the model is then employed to predict interactions between ligands (*e.g.* IGF-I and –II, insulin) and their corresponding receptors (*e.g.* IGF-IR, IGF-IIR and IR) under various physiological conditions through parametric and sensitivity studies.

## Results and Discussions

### Model calibration

Our computational model involves 23 parameters. Fortunately, most of these parameters may be obtained from well-documented experimental and theoretical studies, as detailed in Table I Tables.

Table. However, due to the paucity of direct quantitative measurements in tissues, we must estimate some of the model parameters, specifically the half-life of free IGF and IGFBP within tissue, and the discontinuity in the concentration of macromolecules between the synovial fluid and the tissue's external surface. Moreover, we make the following assumptions.

- The total receptor concentration (*c_RT0_*) in cartilage (with respect to the whole cartilage volume) is estimated to be 0.6 nM [22], but the greatest uncertainty relates to the distribution of different types of receptors on the surface of a tissue cell (*i.e.* chondrocyte). As a first estimate, we assume IGF-IR, IGF-IIR and IR are equally distributed on the surface of a tissue cell (*i.e. c_IR0_*  =  *c_IIR0_*  =  *c_R0_*), although this assumption will be examined in a parametric study.
- As IGFBP and SC have similar molecular weights, we assume that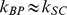.
- The insulin concentration in synovial fluid is assumed to be similar to that in human plasma (*i.e.* 0.2–0.8 nM in human plasma [61]). The effect of varying this insulin concentration will be tested in a parametric study.

**Figure 3:**
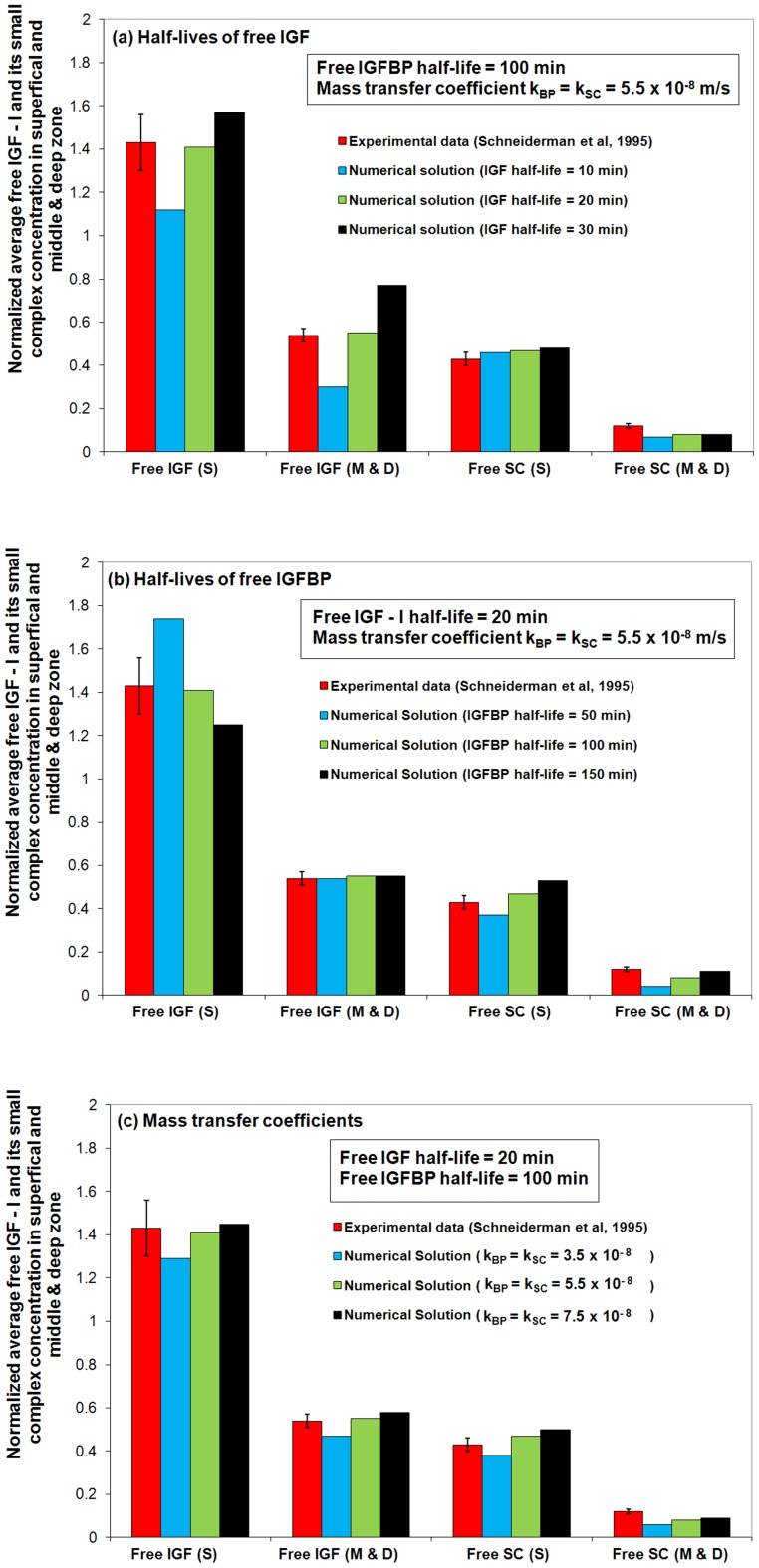
Comparison of the numerical predictions to the experimental data from Schneiderman*et al* (**1995**) [**58**] **.** The steady-state free IGF-I and its small complex concentrations in cartilage superficial zone (S) and middle & deep zone (M & D) are normalized to their synovial fluid concentrations. It can be seen that the experimental results are described remarkably well by a set of parameters, *i.e.*, free IGF half-life  = 20 min, free IGFBP half-life  = 100 min and mass transfer coefficient *k_BP_*  =  *k_SC_*  = 5.5×10^−8^ m/s.

Schneiderman *et al* [58] experimentally studied the concentration and molecular size distribution of IGF-I and its complexes in human synovial fluid and cartilage. Human synovial fluid and femoral heads were obtained from both male and female patients (age range 20–90). The concentrations of free IGF-I and its small binary complex (SC) in synovial fluid, cartilage surface layers (approximately 0.2 mm thick) and the remainder (“middle and deep” zone of the cartilage) were estimated using ultrafiltration membranes (20–100 kDa) followed by a radioimmunoassay of each fraction. The results showed significantly higher concentrations of free IGF-I and its small complex in the ‘superficial zone’ (S) of the tissue, relative to that in the ‘middle and deep’ zone (M & D) of the tissue (see Figure 3). Most interestingly, it was also observed that the free IGF-I concentration in the superficial zone is over 40% *higher* than that in synovial fluid. It is noted that a single species diffusion model does not predict this finding (assuming negligible production of the species within the tissue, one would expect concentrations in the tissue to be less than or equal to the synovial fluid concentrations).

In relation to the current study, the experimental observations reported by Schneiderman *et al* (1995) can be used to estimate the unknown model parameters. That is, we will now proceed to optimize unknown model parameters (specifically optimize the half-lives of two IGFs and their IGFBPs (the same half-life for both groups of IGFBPs), and the mass transfer coefficients of IGFBPs and small complexes between the synovial fluid and cartilage external surface) so as to achieve a best match to the above-mentioned experimental observations.

In plasma, the half-life of free IGF is 10–15 minutes [62] while IGFBP has a longer half-life of about 30–90 minutes [32]. In addition, previous studies on transport of ^14^C-mannital across specific peritoneal tissue surfaces in the rat showed that estimated mass transfer coefficient in liver, stomach, intestines, colon and uterus is around (1∼40)×10^−8^ m/s [63]. This valuable information provides a touchstone for our model calibration. Figure 3 presents the results of the experimental data fitting, which is focused on the half-lives of free IGF and IGFBP and the mass transfer coefficients of IGFBP and small complex (SC) (*i.e.* 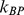
and 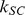). Also included in Figure 3 for comparison, are the experimental results of Schneiderman *et al.* [58]. It is found that these experimental results can be described by the model using IGF-I t_1/2_  = 20 min, IGFBP t_1/2_  = 100 min for half-lives in cartilage and a mass transfer coefficient *k_BP_*  =  *k_SC_*  = 5.5×10^−8^ m/s (which is within the range of values reported above). Note, this single set of parameters can simultaneously reproduced the experimental observations of depth dependent free IGF-I and SC distributions.

As shown in Figure 3a, the overall steady-state free IGF-I uptake is mainly governed by its half-life within the tissue – the longer the half-life of free IGF, the higher the free IGF-I concentration throughout the tissue. In contrast, the IGF half-life appears to have limited influence on free SC uptake. Figure 3b shows that the steady-state free IGF-I concentration in the tissue superficial zone is strongly influenced by the half-life of IGFBP. The faster the degradation of free IGFBP, the greater the release of free IGF from the small complex. This increases the free IGF in the tissue superficial zone. A shorter half-life of IGFBP reduces the distance the free SC is transported into the deeper regions of the cartilage.

A lower mass transfer coefficient means that less free IGFBP and SC in the synovial fluid manages to penetrate the surface of the cartilage tissue per unit time, and will result in a lower IGF and SC concentrations in the tissue. It can be seen from Figure 3c that the model results fit the experimental data reasonably well when *k_BP_*  =  *k_SC_*  = 5.5×10^−8^ m/s. The outcome of data fitting is encouraging, though it is acknowledged that experimental data is limited, and the model clearly needs to be further reassessed in the light of additional experimental data sets.

**Figure 4:**
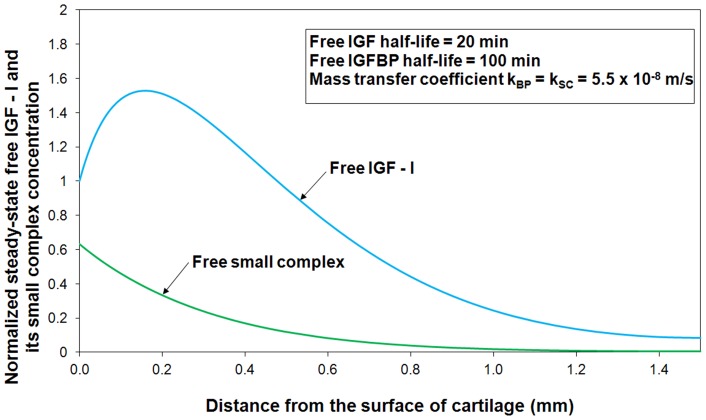
Diffusion of free IGF-I and–II, insulin, two functional groups of IGFBPs and their complexes from synovial fluid into cartilage. The free IGF-I and its complex concentrations are normalized to their respective concentrations in synovial fluid.

By employing model parameters estimated from data fitting (Figure 3), the estimated steady-state free IGF-I and SC concentration profiles throughout the tissue are shown in Figure 4. The calculated concentration of free IGF-I and its complex are normalized to their respective concentrations in synovial fluid. The numerical results show that there is a significantly higher concentration of free IGF-I in the superficial zone (0–0.2 mm) of the cartilage, which is well above the ‘source concentration’ of free IGF-I in synovial fluid. This computational result is consistent with the experimental observations [58]. Maximum free IGF concentration (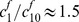) occurs at around 0.2 mm from the tissue's external surface, but then decreases with increasing depth in the tissue, reaching about 10% of the synovial fluid IGF concentration in the deepest regions of the cartilage (*i.e.* 1.5 mm). The model predicts that the free SC concentration immediately inside the tissue surface is around 60% of that in synovial fluid. The results in Figure 4 are sensible because of the selective degradation of the IGFBPs by proteases, which results in an internal maximum normalized free IGF-I ratio inside the cartilage itself. Our recent study [33] theoretically demonstrated that reversible binding (i.e. IGF-I and IGFBP3) plus preferential degradation of free IGFBP3 significantly increases of IGF-I uptake into the cartilage tissue. We note that our results for the complete IGF system, shown in Figure 4, are consistent with our previous findings.

The calibrated model can now be employed to predict the concentration of ligand/receptor complex distribution throughout the tissue for a range of perturbations to this system. The focus is on the ligand/receptor complex as a model output as the binding of IGF-I, -II and insulin to IGF-IR receptors initiates intracellular signaling and the subsequent cell response.

**Figure 5:**
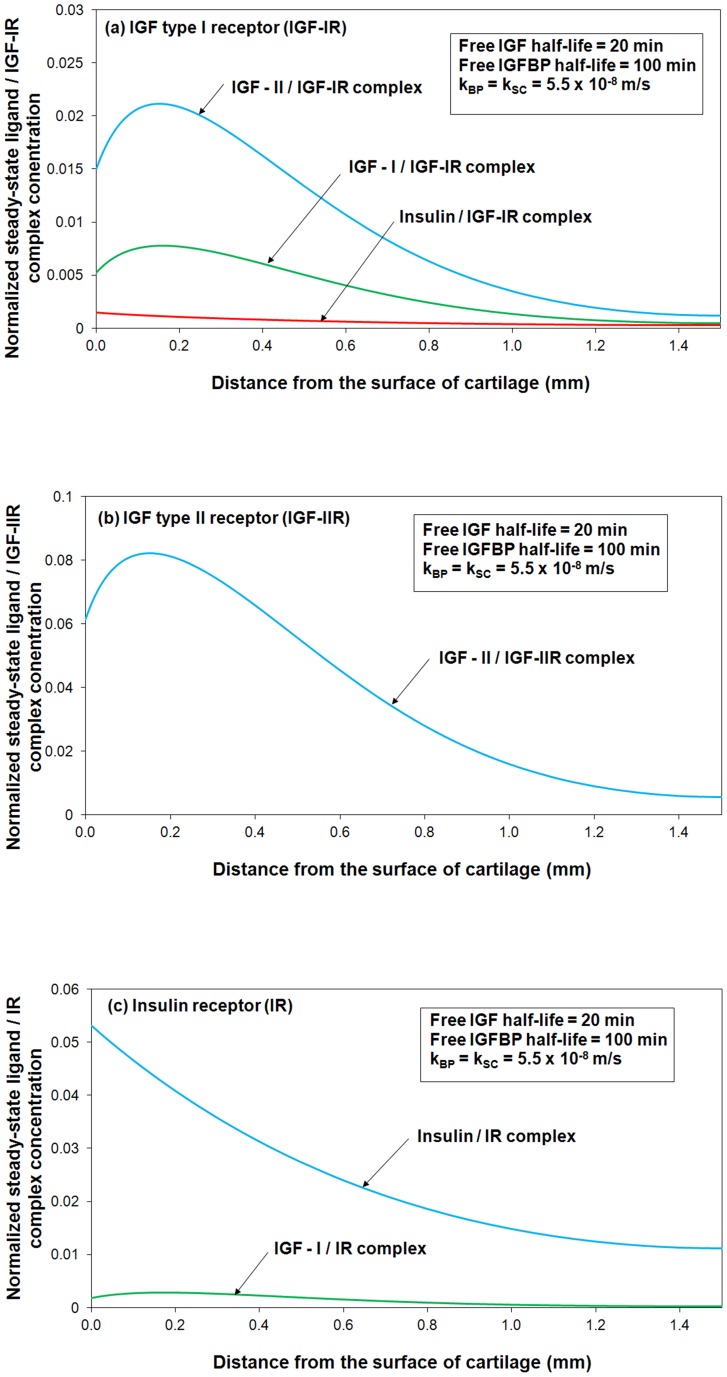
Steady-state concentrations of ligands (*i.e.* IGF-I, IGF–II and insulin) and their corresponding receptor (*i.e.* IGF-IR, IGF-IIR and IR) complexes within the cartilage. The calculated complex concentrations are normalized to the total receptor concentration (*i.e. c_RT0_*  =  0.6 nM).

Figure 5 shows the steady-state concentrations of ligands (*i.e.* IGF-I, IGF–II and insulin) and their corresponding receptor (*i.e.* IGF-IR, IGF-IIR and IR) complexes within the cartilage. It can be seen that the steady-steady ligand/receptor complex concentration is much higher in the superficial zone compared to that in M & D zone. The numerical outcomes are consistent with various studies which postulated that articular cartilage superficial zone represents an important signaling centre that is involved in regulation of tissue development and growth [64], [65]. For example, the experimental studies of Hayes *et al*. indicated that the tissue near the articular surface may contain a population of progenitor cells that are responsible for the appositional growth during early development of the tissue instead of interstitial growth [65]. Figure 5a is also shown that only a small portion of cell surface receptors are bound to IGFs. The simulation outcomes indicated that most of the cell surface receptors are inactive in normal conditions. Indeed, based on the experimentally measured receptor concentration in cartilage and binding affinity of IGF to IGF-IR which are shown in Table 1, and our model, we can for the first time confidently predict the what the occupancy for IGF-IR actually is in the tissue.

**Table 1:** Parameters used for fitting equations (27)–(34) to the data of Schneiderman *et al* [58].

Parameter	References and comments
Diffusion coefficient of IGF-I and –II (7.6 kDa) (*D_IGF_*)	(2–4)×10^−7^ cm^2^/s [75]
Diffusion coefficient of insulin (5.8 kDa) (*D_INS_*)	2×10^−7^ cm^2^/s [76]
Diffusion coefficient of IGFBP (*D_BP_*)	(0.6–1.3)×10^−7^ cm^2^/s [22]
Diffusion coefficient of small complex (*D_SC_*)	(0.6–1.3)×10^−7^ cm^2^/s [22]
Free IGF-I concentration in human synovial fluid (*c^f^_10_*)	0.066 nM [19], [58]
Free IGF-I small complex concentration in human synovial fluid (*c^f^_SC110_*)	2.6 nM [58]
Insulin concentration in human synovial fluid (*c^f^_30_*)	0.2–0.8 nM in serum [61]
Total receptor concentration (*c_RT_*)	0.6 nM [22]
Equilibrium dissociation constant for IGF-I and IGFBPs 1–5 (*K_D11_ = k*_−11_/*k*_+11_)	4.8 nM [43]
Equilibrium dissociation constant for IGF-II and IGFBPs 1-5 (*K_D21_ = k*_−21_/*k*_+21_)	5.2 nM [43]
Equilibrium dissociation constant for IGF-II and IGFBP6 (*K_D22_ = k*_−22_/*k*_+22_)	5.7 nM [43]
Association rate constant for IGF-I and –II and IGFBPs (*k*_+11_, *k*_+21_ and *k*_+22_)	(0.1–9)×10^5^ M^−1^s^−1^ [43]
Equilibrium dissociation constant for IGF-I and IGF-IR (*K_D11R_ = k*_−11*R*_/*k*_+11*R*_)	1.4 nM [71]
Equilibrium dissociation constant for IGF-II and IGF-IR (*K_D21R_ = k*_−21*R*_/*k*_+21*R*_)	(2∼15)×*K_D11R_* [32]
Equilibrium dissociation constant for insulin and IGF-IR (*K_D31R_ = k*_−31*R*_/*k*_+31*R*_)	(50∼100)×*K_D11R_* [32], [77]
Equilibrium dissociation constant for IGF-II and IGF-IIR (*K_D22R_ = k*_−22*R*_/*k*_+22*R*_)	0.017∼0.7 nM [32]
Equilibrium dissociation constant for insulin and IR (*K_D33R_ = k*_−33*R*_/*k*_+33*R*_)	0.1 nM [47]
Equilibrium dissociation constant for IGF-I and IR (*K_D13R_ = k*_−13*R*_/*k*_+13*R*_)	(50∼100)×*K_D33R_* [32], [77]
Associate rate for IGF and receptors (*k*_+11*R*_, *k*_+21*R*_, *k*_+31*R*_, *k*_+22*R*_ and *k*_+11*R*_)	(1.8–4.5)×10^5^ M^−1^s^−1^ [22]
Receptor internalization rate (*k*_10_, *k*_20_, *k*_30_)	(0.5–3)× *K_D11R_*×*k_+_*_11*R*_ [22]

### Parametric studies

#### The ratios of IGF-I, IGF-II and insulin

A poorly understood but apparently important mechanism worthy of further investigation is the ratio between IGF-I and IGF-II within tissue. Recent evidence has indicated that high IGF-II concentration in circulation may lead to an increased risk for developing breast, prostate, colon and lung cancer [25]. There is reportedly a 4-fold increase of the total IGF-I/IGF-II ratio in OA synovial fluid [26].

**Figure 6:**
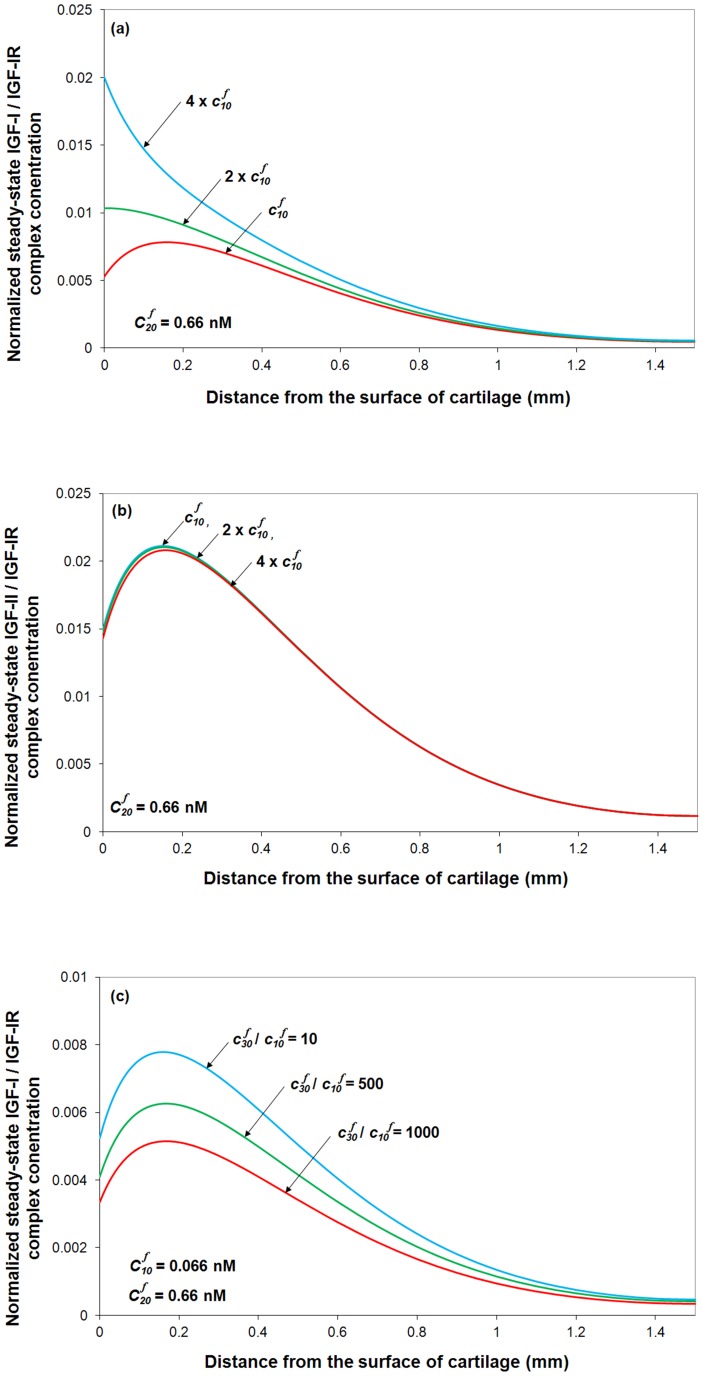
The effects of the ratios of IGF-I, IGF-II and insulin on normalized steady-state IGF-IR complex concentration. The calculated complex concentrations are normalized to total receptor concentration (*i.e. c_RT0_*  = 0.6 nM). Free IGF half-life  = 20 min, free IGFBP half-life  = 100 min, mass transfer coefficient *k_BP_*  =  *k_SC_*  = 5.5×10^−8^ m/s, and 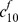 = 0.066 nM.

By fixing free IGF-II and IGFBP concentrations in synovial fluid (*i.e.* 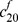 = 0.66 nM) and varying synovial fluid IGF-I concentration, the model predicts that in OA condition, the 4-fold increase of IGF-I significantly increases IGF-I/IGF-IR complex concentration in the the superficial zone by around 30%, whilst has little impact on IGF-II/IGF-IR complex concentration (see Figure 6a and Figure 6b). Presumably this would enhance IGF-I mediated biological activity but have little influence on IGF-II induced cellular activities. Further, it can be seen that only very high concentration of insulin can influence IGF-I/IGF-IR complex concentration (Figure 6c). Most importantly, it seems only 10% IGF-IR is complexed with ligand with around 1% with IGF-I.

**Figure 7:**
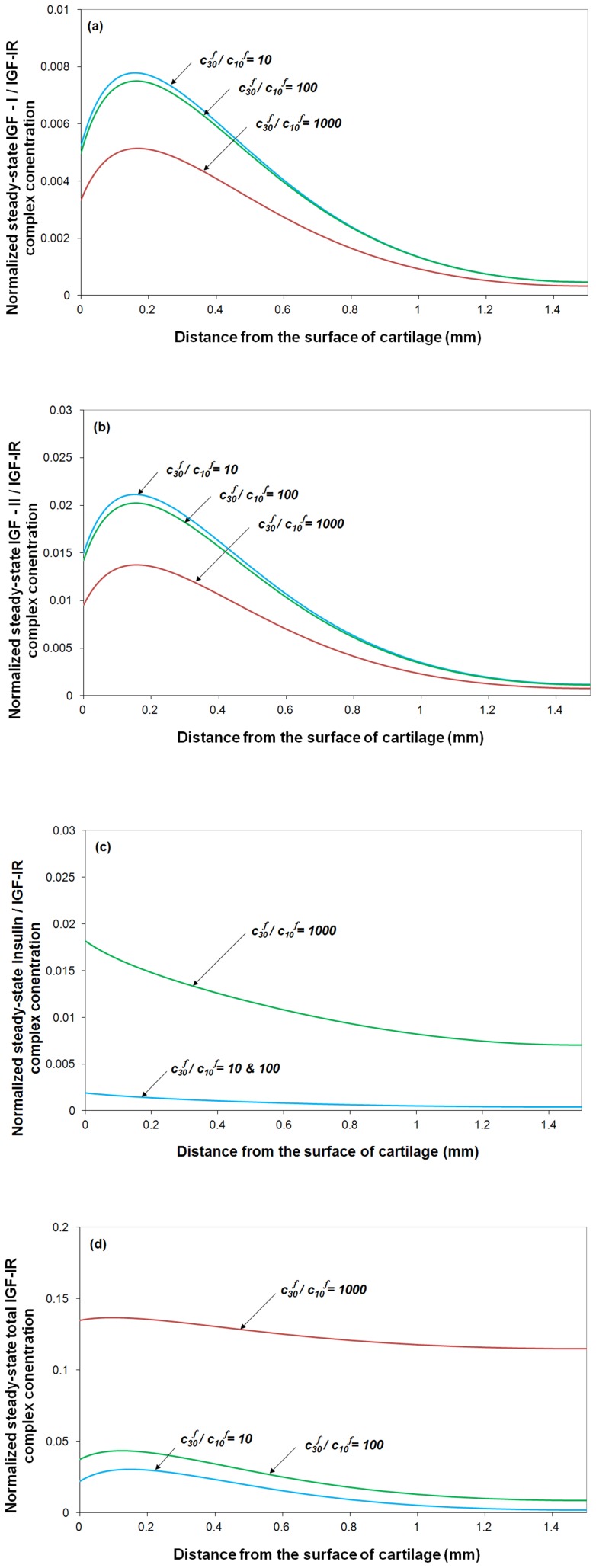
Steady-state IGF-I/IGF-IR, IGF–II/IGF-IR, insulin/IGF-IR and total IGF-IR complex concentration in cartilage under various insulin to IGF-I ratios in synovial fluid. The calculated complex concentrations are normalized to total receptor concentration (*i.e. c_RT0_*  =  0.6 nM). Free IGF half-life  =  20 min, free IGFBP half-life  =  100 min, mass transfer coefficient *k_BP_*  =  *k_SC_*  = 5.5×10^−8^ m/s, and 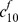 = 0.066 nM.

Turning our attention to the influence of insulin on the IGF-I/IGF-IR, IGF-II/IGF-IR and insulin/IGF-IR formation, Figure 7 suggests that insulin only has an effect at very high insulin concentrations (*i.e.* 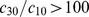, 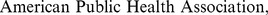  = 0.066 nM) due to its relatively low binding affinity of insulin for IGF-IR. The computational model suggests that a higher insulin concentration (*i.e.* 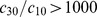) could potentially decrease IGF-I/IGF-IR and IGF-II/IGF-IR complex concentration in the cartilage superficial zone (Figure 7a-b) but significantly increase insulin/IGF-IR complex formation (Figure 7c) and the total IGF-IR complex formation throughout the tissue (Figure 7d). The normal range of concentration of insulin is 0.2–0.8 nM in human plasma [61]. Any significant difference in unlikely to be seen without at least an order of magnitude increase in plasma concentration of insulin. Although IGF-IR is highly specific to IGF-I and –II, insulin can still activate IGF-IR at higher tissue concentrations (*i.e.* >10 nM or 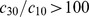) [66]. A recent study on the effect of insulin on proteoglycan synthesis in porcine articular cartilage explants showed that insulin at 10 nM increased proteoglycan synthesis by 240% and inhibited the IL-1 induced proteoglycan catabolism [67].

#### The ratios of two functional IGFBP groupings

**Figure 8:**
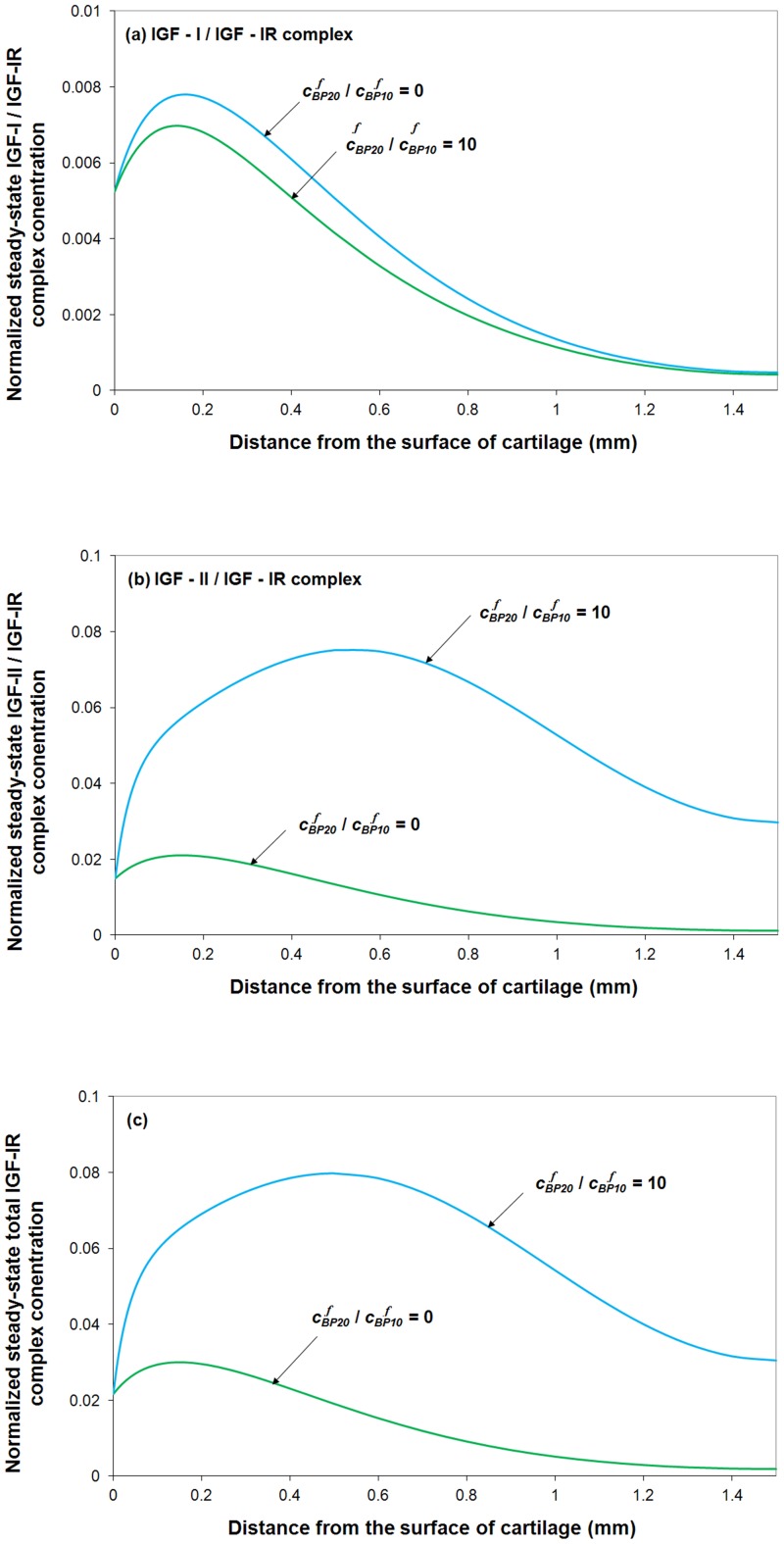
Steady-state IGF-I/IGF-IR, IGF-II/IGF-IR complex and total IGF-IR concentration under various ratios of two functional IGFBP groupings in synovial fluid. The calculated complex concentrations are normalized to total receptor concentration (*i.e. c_RT0_*  = 0.6 nM). Free IGF half-life  = 20 min, free IGFBP half-life  = 100 min, mass transfer coefficient *k_BP_*  =  *k_SC_*  = 5.5×10^−8^ m/s, and 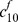 = 0.066 nM.

Research on IGFBP-6 is relatively limited compared to research on IGFBPs 1–5. As far as known, IGFBP6 preferentially binds IGF-II compared with IGF-I [40], [41], [42]. The IGFBP6 content within a tissue varies between species, *e.g.*, IGFBP6 is one of the major IGFBPs in bovine cartilage [44], and yet its concentration is too small to be detected in normal human cartilage [26]. While the knowledge of IGFBP6 is relatively limited, using the computational model we can explore the functional role of IGFBP6 in modulating IGF bioavailability in tissue. Figure 8 shows the effect on the steady-state total IGF-IR complex concentration in the cartilage of varying the ratio of the two IGFBP functional groups in the synovial fluid (*i.e.* 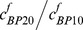). As IGF-I and –II, and the two functional groups of IGFBPs and their complex are in reversible equilibrium in synovial fluid, a higher 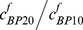 ratio (*e.g.* 
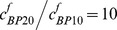
) indicates that much more IGF-II is transported into the cartilage, in comparison to IGF-I. Ultimately this leads to a decrease in the steady-state IGF-I/IGF-IR complex concentration throughout the tissue (Figure 8a) but a very significant increase of the IGF-II/IGF-IR and total IGF-IR complex concentration (Figure 8b–c).

#### The degradation of two functional IGFBP groupings

**Figure 9:**
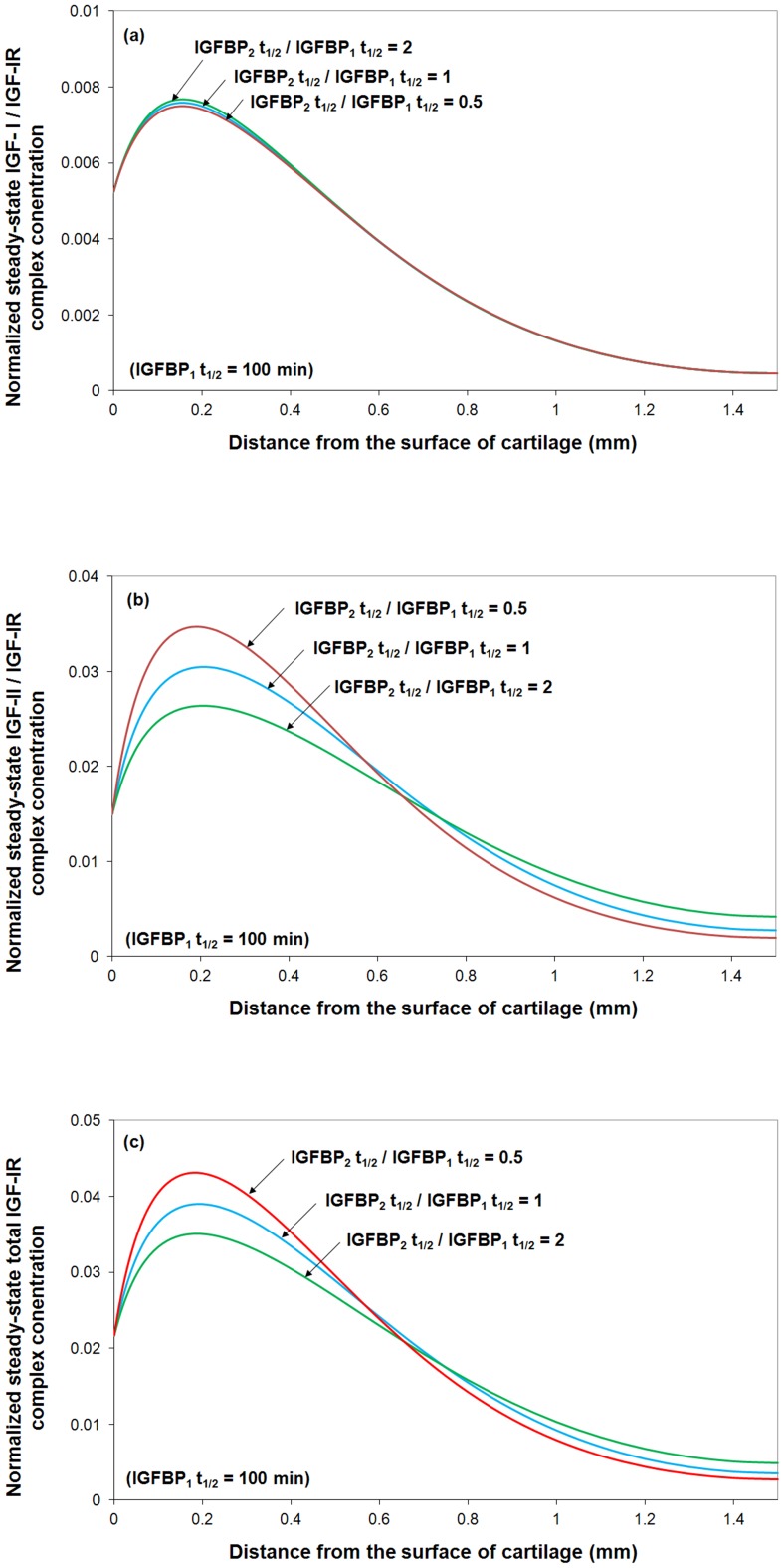
Steady-state IGF-I/IGF-IR, IGF-II/IGF-IR and total IGF-IR complex concentration under various ratios of the half-life of two functional IGFBP groupings. **The calculated complex concentrations are normalized to total receptor concentration** (***i.e. c_RT0_*** **= 0.6 nM**)**.** Free IGF half-life  = 20 min, 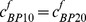, mass transfer coefficient *k_BP_*  =  *k_SC_*  = 5.5×10^−8^ m/s, and 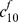 = 0.066 nM.

IGFBP6 regulates the biological action of IGF-II [68]. Studies have also shown that different proteases preferentially target different IGFBPs for degradation. For example ADAM 12-S degrades only IGFBP3, leaving IGFBP6 [34]. By fixing the half-life of the first functional group of IGFBP (*i.e.* IGFBPs 1–5) and varying the half-life of the second functional group of IGFBP (*i.e.* IGFBP6), the model results shown in Figure 9 demonstrate that IGFBP6 is capable of regulating the total IGF-IR complex concentration via proteases mediated degradation of IGFBP6. There is little data for humans, but during ‘reposition loading’ on rabbit mandibular cartilage to adjust a occlusional defect, IGFBP6 expression underwent a 3-fold change in expression over the 35 day load period [69].

Most interestingly, although the half-life of IGFB6 has little influence on IGF-I/IGF-IIR complex formation, the IGFBP6 degradation has a spatial dependent effect on the IGF-II/IGF-IR and the total IGF-IR complex concentrations, *i.e.*, a higher degradation rate of IGFBP6 has obviously positive effect on IGF-II/IGF-IR and total IGF-IR complex formation in the superficial zone but some negative effects in the M & D zone.

#### The ratio of IGF-IR, IGF-IIR and IR on the surface of a tissue cell

The kinetics of competition of ligands (*e.g*. IGF-I and –II, insulin) for cell surface receptors (*e.g.* IGF-IR, IGF-IIR and IR) has been intensively studied for several decades [33], [56]–[58]. Using cartilage tissue from human knee joints as an example, experimental studies showed that there are approximately 18,000 chondrocytes /mm^2^ per 350 µm (thick) on average [70], and about 20,000 receptors per cell [71].However, there is little experimental information about the actual distribution between IGF-IR, IGF-IIR and IR on the surface of a cell. Furthermore, this distribution is likely to differ from species to species as well as from tissue to tissue.

**Figure 10:**
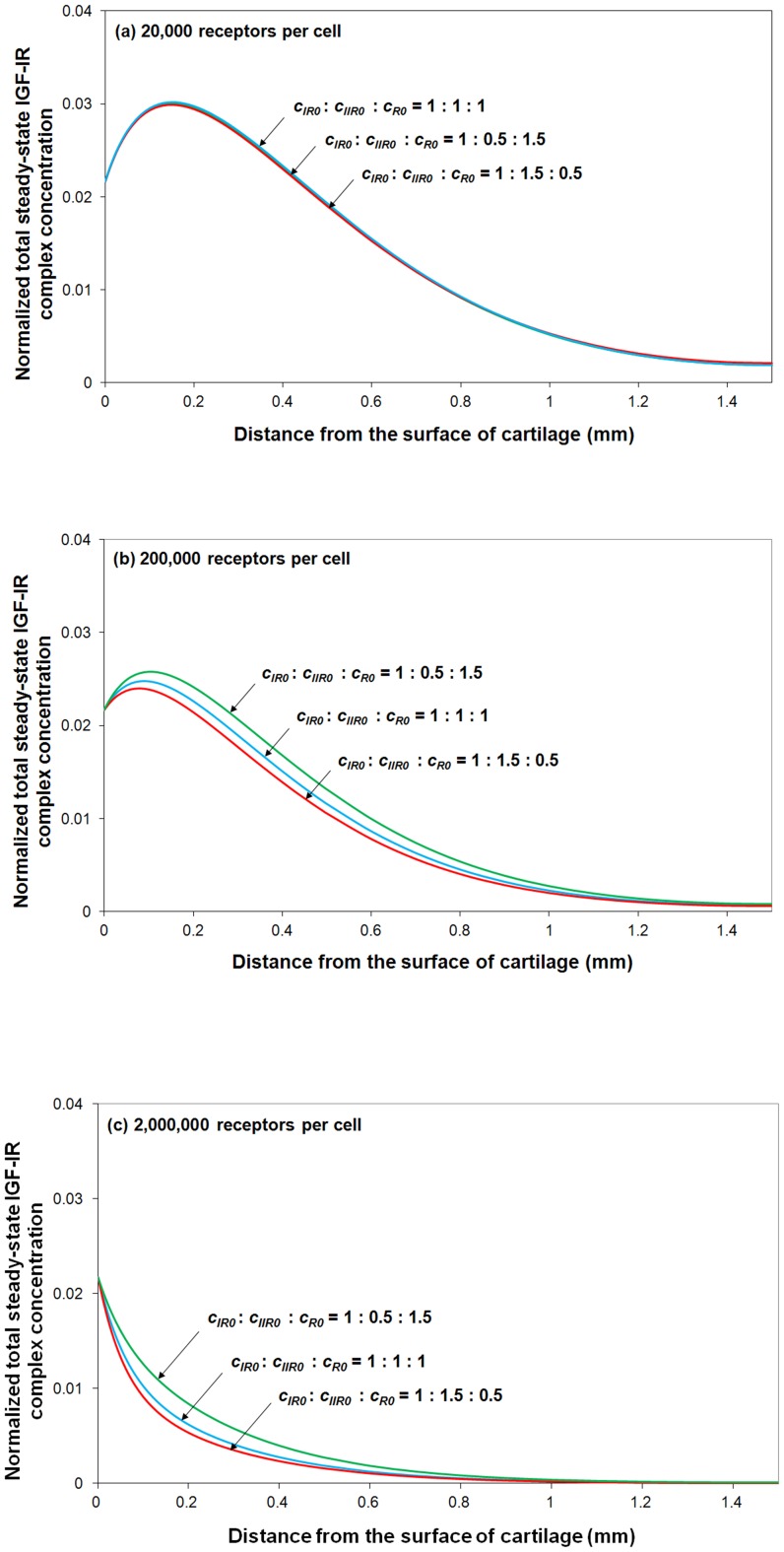
Normalized steady-state total IGF-IR complex concentration under various distribution of IGF-IR, IGF-IIR and IR receptors. The calculated complex concentration is compared to total receptor concentration. Free IGF-I half-life  = 20 min, free IGFBP half-life  = 100 min, mass transfer coefficient *k_BP_*  =  *k_SC_*  = 5.5×10^−8^ m/s.

Thus, a series of parametric studies are carried out here to investigate the effect of this receptor distribution and receptor density on IGF-IR complex formation. By varying different types of receptor distribution, while fixing the total number of cells within a tissue, it can be seen (in Figure 10) that receptor distribution has little influence on total IGF-IR complex concentration when the number of receptors per cell is relatively low (*e.g.* 20,000 receptors per chondrocyte in human cartilage). However, this distribution has some effect for a tissue with much higher receptors per cell (*i.e.* >200,000 receptors per cell). It is thought that the inhibitory effects of IGFBPs on IGF-I and –II are largely due to the higher affinity of the two IGFs for IGFBPs than that of IGF-IR [1], [35]. Our simulation results show that this “blocking” capability of IGFBPs gradually deteriorates with the increase in the receptor concentration (relative to the IGFBP concentration).

By fixing the fraction of IGF-IR (*i.e.* 33% of total receptor types per cell), it can be seen from Figure 10b–c that the total IGF-IR complex concentration changes inversely with the IGF-IIR fraction. The results presented here demonstrate that IGF-IIR could influence the complex formation between IGF-II and IGF-IR *e.g*. by functioning as an IGF-II clearance receptor.

Finally, Figure 10c indicates that at very high receptor concentration, most of the IGFs and insulin are consumed within the tissue superficial zone, and thereby have little chance of reaching into the deep region of the tissue.

#### Ligand / receptor half-life

**Figure 11:**
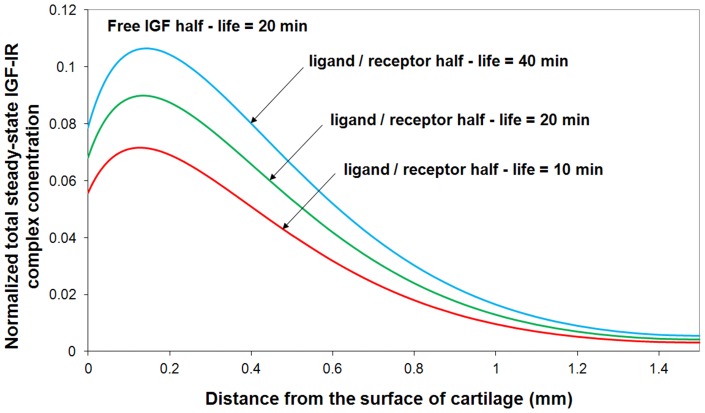
Normalized steady-state total IGF-IR complex concentration under various ligand/receptor half-lives. The calculated IGF-IR complex concentration is compared to total receptor concentration. Free IGF-I half-life  = 20 min, free IGFBP half-life  = 100 min, mass transfer coefficient *k_BP_*  =  *k_SC_*  = 5.5×10^−8^ m/s.

IGF signaling depends on the conversion of the interaction between ligand (IGF-I, IGF-II and insulin) and IGF-IR into changes in cell biology. As shown in Figure 11, in this study, we theoretically studied the effects of ligand / IGF-IR complex half-lives (i.e. the receptor internalization rate following the binding of ligand to IGF-IR) on total steady-state IGF-IR complex concentration. By fixing the half-life of free IGF (i.e. t_1/2_  = 20 min), it is demonstrated that chondrocytes can regulate their own exposure to free IGF by controlling the ligand internalization rate, *k_0_*. These simulation results are consistent to other relevant research studies [72], [73].

#### IGF-IIR concentration

**Figure 12:**
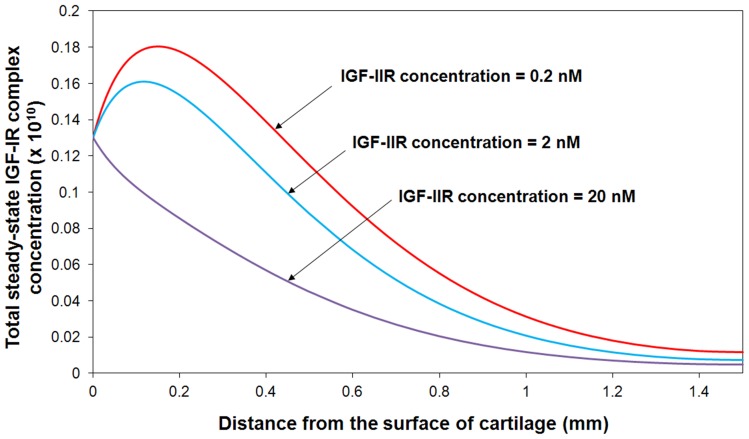
Steady-state total IGF-IR complex concentration under various IGF-IIR concentrations. The calculated IGF-IR complex concentration is compared to total receptor concentration. Free IGF-I half-life  = 20 min, and mass transfer coefficient *k_BP_*  =  *k_SC_*  = 5.5×10^−8^ m/s.

Figure 12 shows a strong connection between IGF-IIR concentration and IGF-IR signaling through modifying total IGF-IR complex concentration. The results demonstrate that IGF-IIR could function as a “clearance receptor” by removing IGF-II from the matrix environment. These observations are consistent to the research studies on the roles of the IGF system in cancer growth and metastasis which indicate that IGF-IIR are negative effectors that mediate the IGF-IR signaling and function [14].

#### Sensitivity analysis

A sensitivity analysis can be used to identify the dominant parameters in the IGF system affecting a state variable of interest. In our case the state variable of interest is the total IGF-IR complex concentration as it is this concentration regulating the subsequent intra-cellular signaling. Identifying the parameters to which the system is most sensitive helps focus future experiments, as reducing the uncertainty in these parameters has most impact on reducing model uncertainty. Further, the tissue (or clinician through the administration of drugs) may target the parameters identified by a sensitivity analysis to efficiently control the system. Indeed, if there are several parameters that strongly influence the system, then all these parameters may need to be controlled simultaneously to control the system. We return to this point later in the discussion.

The basis of a sensitivity analysis is systematically varying one parameter at a time and observing the corresponding change in the system output of interest. This implies performing the sensitivity analysis about a ‘base’ set of model parameters that represents the operating point of the system. For the operating point, here we use the model parameters optimised to reproduce the cartilage experimental data of Schneiderman *et al* (1995) [58] (*i.e.* free IGF-I half-life  = 20 min, free IGFBP half-life  = 100 min, mass transfer coefficients *k_BP_*  =  *k_SC_*  = 5.5×10^−8^ m/s). As for the insulin, its concentration in normal human plasma (*i.e.* 0.5 nM) [61] is treated as the base value for this sensitivity analysis. However, it is important to note that due to system non-linearities, the set of parameters to which a system is most sensitive may change if a new system operating point (and set of base parameters) were to be chosen.

We start with the calculation of the ‘base’ amount of total IGF-IR complex within the cartilage using COMSOL sub-domain integration (*i.e. I*_IGF-IR complex_base_). Then, the value of each parameter is systematically varied ranging over six orders of magnitude (from 0.001∼1000) of its base value to explore the change of total IGF-IR complex concentration (*i.e. I*_IGF-IR complex_) with respect to *I*_IGF-IR complex_base_ in the superficial zone, M & D zone and the overall tissue respectively.

**Figure 13:**
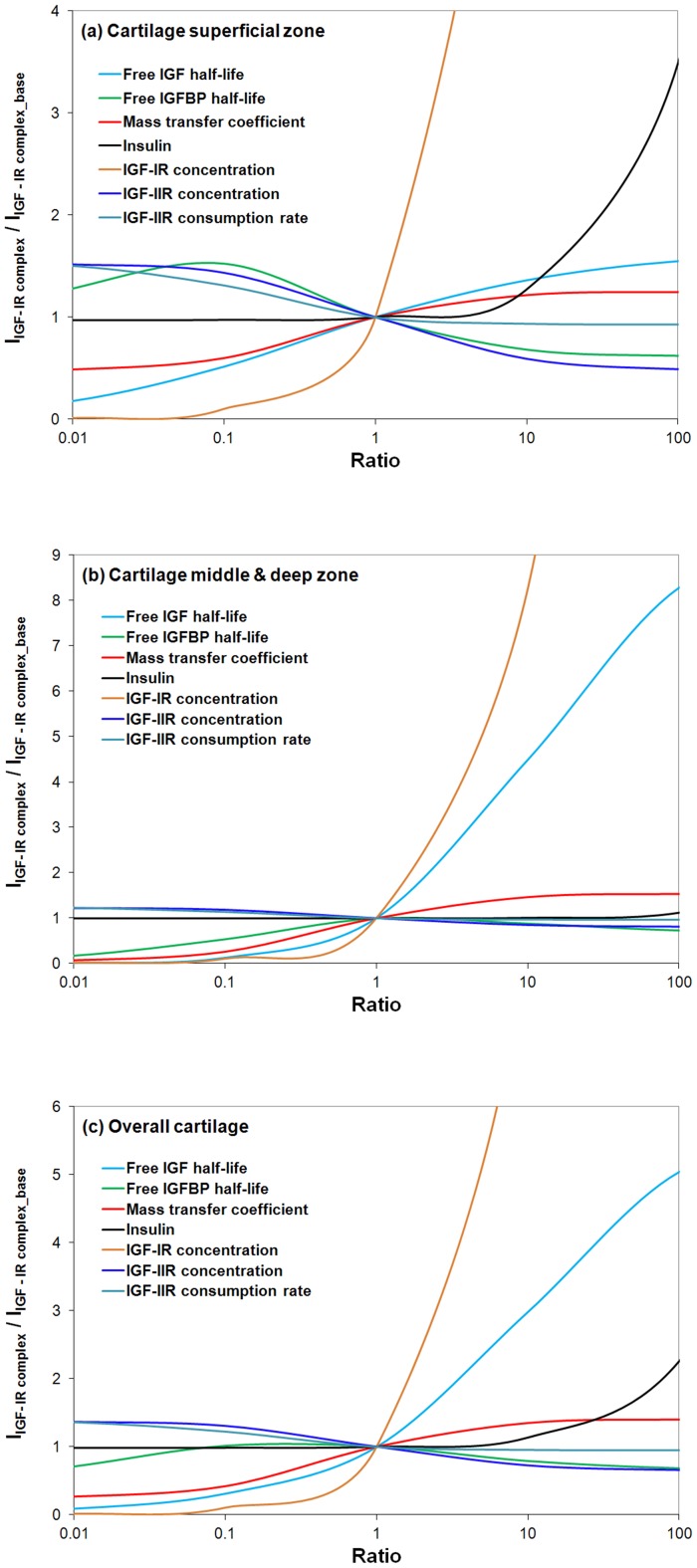
Sensitivity analysis of model parameters on steady-state total IGF-IR complex concentration integrated over cartilage superficial zone, middle & deep zone and overall tissue respectively. The calculated sub-domain integration (*i.e.* I_IGF-IR complex_) is compared to its base value (*i.e.* I_IGF-IR complex-base_). The base values of model parameters (*i.e.* half-life of free IGF, IGFBP and ligand/receptor, mass transfer coefficient and insulin) are obtained from model calibration using the experimental data from Schneiderman *et al* (1995) [58] (*i.e.* free IGF-I half-life  = 20 min, free IGFBP half-life  = 100 min, IGF-IIR concentration  = 20 nM, mass transfer coefficient *k_BP_*  =  *k_SC_*  = 5.5×10^−8^ m/s) while the base value of insulin concentration  = 0.5 nM reported in normal human serum [61].

**Figure 14:**
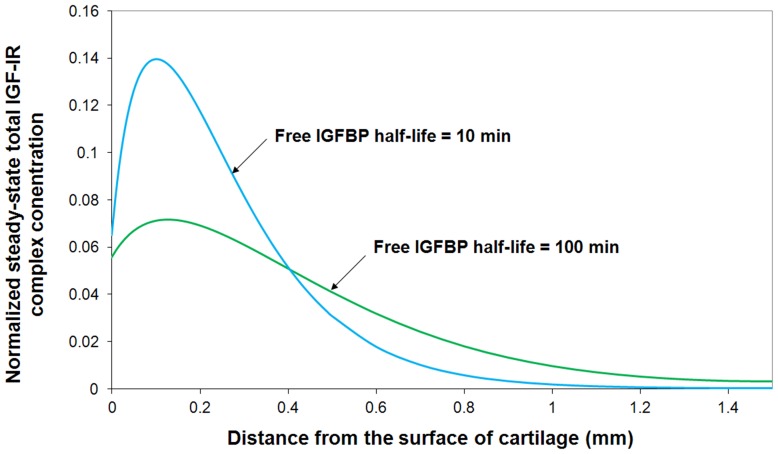
Normalized steady-state total IGF-IR complex concentration when free IGFBP half-life is equal to 10% of its base value (**i.e. 10 min**)**.** The calculated IGF-IR complex concentration is compared to total receptor concentration. Free IGF-I half-life  = 20 min, and mass transfer coefficient *k_BP_*  =  *k_SC_*  = 5.5×10^−8^ m/s.

In this study, we mainly focus on some of the model parameters which are not well understood in the cartilage so far (*i.e.* half-lives of free IGF and IGFBP within the tissue, mass transfer coefficient of IGFBP, IGF-IR concentration, IGF-IIR concentration and its consumption rate, and concentration of insulin). The results appear in Figure 13. IGF-IR concentration apparently is the most sensitive parameter. It indicates that the most effective way of controlling the IGF system is through activating and deactiving of IGF-IR. The half-life of IGF is also shown to be one of the most critical parameters governing the concentration of IGF-IR complex within the tissue and its effect appears to be strongly depth dependent. For example, a 10-fold increase of the base value of free IGF half-life could potentially increase total IGF-IR complex formation by around 36% in the superficial zone, 450% in M & D zone and 300% throughout the tissue. Further, our results suggest that an optimal IGFBP degradation rate may be different in the superficial zone and M & D zone if the goal is to maximize IGF-IR complex formation. That is a trade-off exists between maximizing superficial versus M & D zone receptor complex formation. Interestingly, the calibrated model (and presumably the cartilage) is operating with an IGFBP degradation rate that gives the optimal overall (tissue averaged) receptor complex concentration. That is, the magnitude and direction of IGFBP degradation gradient offers the control over the system. As shown in Figure 13a, one tenth of the base value of IGFBP half-life appears to be the optimal half-life of IGFBP in superficial zone (increase IGF-IR complex by 52% in superficial zone) whilst the base value of IGFBP half-life appears to be the optimal value in M & D zone. The depth dependent total IGF-IR complex concentration profile under the optimal half-life of free IGFBP in superficial zone (i.e. 10 min which is 10% of its base value) is shown in Figure 14. It can been seen that this optimal half-life of free IGFBP leads to an significant increase of total IGF-IR complex concentration in superficial zone but a relatively lower complex concentration in M & D zone. The implication of these results is that cartilage could optimize the exposure of IGF-IR to IGF in different regions of the tissue by spatially adjusting the rate of IGFBP degradation (*i.e*. the chondrocytes could ‘tune’ their exposure to IGF by adjusting the rate of IGFBP protein degradation). Further, a balance between IGF signaling and controlling this signaling is of importance for chondrocytes to maintain tissue homeostasis. Figure 13 shows that there is a generally positive correlation between the mass transfer coefficient and the total IGF-IR complex concentration. A mass transfer coefficient less than the base value (*i.e.* 5.5×10^−8^ m/s) could potentially decrease total IGF-IR complex concentration but has little influence once the value is greater than its base value. That is, the mass transfer coefficient is not one of the major parts of the control system used by chondrocytes to tune their exposure to IGF.

Further, it demonstrates that, in comparison to other parameters, IGF-IIR concentration and its rate of consumption are two critical parameters which could significantly influence the total IGF-IR complex concentration. Previous studies have suggested that IGF-IIR functions as a tumor suppressor, while the mutation or loss of IGF-IIR in some human tumors is frequently observed [74]. However, the actual suppressive mechanism of IGF-IIR has not been well understood. Here we demonstrate the ability of IGF-IIR to mediatie the IGF-IR signaling by regulating IGF-IIR concentration or its turnover.

Indeed, our observations gain additional significance following recent findings that new drugs designed to block the IGF-1R receptor have been ineffective in blocking IGF signaling [17]. Allison's suggestion that to block IGF signaling requires a ‘cocktail’ of drugs is consistent with our findings that there are several systems that may independently control the level of IGF-IR complexation. Potential homeostatic feedback systems exerting strong control over IGF-1R signaling are the IGF-IIR concentration and its turnover, and the protease composition in the extracellular environment surrounding the cell, which controls the rate of degradation of IGF and its binding proteins.

Finally, the sensitivity analysis suggests that the formation of IGF-IR complex is generally insensitive to insulin due to its relatively low binding affinity to IGF-IR in comparison to IGFs. However, a significant effect can be seen once the concentration of insulin is over 10 times of the base value in superficial zone where most of the IGFs and IGF-IR complexes in tissue are formed.

## Conclusion

In this study, we have developed a comprehensive mathematical model of the IGF system that may be applied to all tissues (Figure 2). The model is applied here to articular cartilage as appropriate data is available for this tissue to calibrate the model. For this tissue, our main findings are as follows:

- Calibrating the model to reproduce the available experimental data by optimizing over a small subset of model parameters, we have obtained an estimate for the half-lives of IGFs and IGFBPs, mass transfer coefficients of IGFBPs and small complexes within the tissue. Specifically we estimate in cartilage IGF-I t_1/2_  = 20 min, IGFBP t_1/2_  = 100 min and a mass transfer coefficient *k_BP_*  =  *k_SC_*  = 5.5×10^−8^ m/s.
- The model predicts that the distribution of the steady-state concentrations of free IGF-I and -II and their binary complexes are strongly depth-dependent in cartilage, with significantly higher free IGF-I concentration occurring in the tissue superficial zone (0∼0.2 mm), which is well above the free IGF-I concentration in synovial fluid (by around 50%). It is noted that this finding is cannot be reproduced by a simple diffusion model, which predicts all concentrations within the tissue are less than or equal to the concentration in the synovial fluid.
- The half-life of free IGFs govern the steady-state free IGF uptake throughout the tissue, whilst steady-state free IGF concentration in the tissue superficial zone is largely dominated by the half-life of the free IGFBP.
- The majority of IGF molecules from the synovial fluid bind to IGF receptors located in the superficial zone, which leads to the spatial dependent free IGF distribution in cartilage.
- The occupancy of IGF-1R receptors throughout cartilage is low, with more than 95% of these receptors unbound.
- The formation of IGF-IR complex is generally insensitive to insulin in normal conditions. However, insulin concentrations more than 10 times that normal human plasma could significantly enhance overall IGF-IR complex concentration in cartilage.
- Our sensitivity analysis shows that at the normal operating point for cartilage, the receptor occupancy of the IGF-1R receptor is most strongly influenced by the following variables: the half-life of IGFBP, the half-life of free IGF, the IGF-IIR concentration, the IGF-IIR receptor turnover and the mass transport of IGF into the cartilage from the synovial fluid.
- It is likely that the chondrocytes can adjust their expression of proteases to control the half-life of free IGF and its binding proteins in their extracellular environment, and adjust their concentration of IGF-1R and IGF-IIR receptors in the cell membrane, and adjust the rates of receptor turnover. By these means, it is possible for chondrocytes to have some control over their own IGF signalling level within the tissue.

Recent drugs developed to block IGF signaling through its receptor have been disappointing in their therapeutic efficacy. Consequently Allison [17] suggests that a cocktail of drugs is required to block IGF signaling. Our analysis of IGF system is consistent with this view. Potential homeostatic feedback systems exerting strong control over IGF-1R signaling are the IGF-IIR concentration and its turnover, and the protease composition in the extracellular environment surrounding the cell, which controls the rate of degradation of IGF and its binding proteins. We conclude that a systems model of IGF in tissues can assist in developing an understanding of the IGF system that is not possible using experimental methods alone, and that this approach may be useful in assessing the likely efficacy of proposed IGF drug treatments that involve multiple targets.

## References

[1] Durai R, Yang W, Gupta S, Seifalian AM, Winslet MC (2005) The role of the insulin-like growth factor system in colorectal cancer: review of current knowledge International Journal of Colorectal Disease. 20: 203–220. 10.1007/s00384-004-0675-4 15650828

[2] Velloso CP (2008) Regulation of muscle mass by growth hormone and IGF-I. British Journal of Rheumatology 154: 557–568. 10.1038/bjp.2008.153 PMC2439518 18500379

[3] Mohan S, Baylink DJ (2002) IGF-binding proteins are multifunctional and act via IGF-dependent and -independent mechanisms. Journal of Endocrinology 175: 19–31. 12379487 10.1677/joe.0.1750019

[4] D'Ercole AJ, Ye P (2008) Expanding the mind: IGF-I and brain development Endocrinology. 149: 5958–5962. 10.1210/en.2008-0920 PMC2613055 18687773

[5] Foulstone E, Prince S, Zaccheo O, Burns JL, Harper J, et al (2005) Insulin-like growth factor ligands, receptors, and binding proteins in cancer. Journal of Pathology 205: 145–153. 15641016 10.1002/path.1712

[6] Chernausek SD, Backeljauw PF, Frane J, Kuntze J, Underwood LE (2007) Long-Term Treatment with Recombinant Insulin-Like Growth Factor (IGF)-I in Children with Severe IGF-I Deficiency due to Growth Hormone Insensitivity. The Journal of Clinical Endocrinology & Metabolism 92: 902–910. 17192294 10.1210/jc.2006-1610

[7] Juul A (2003) Serum levels of insulin-like growth factors I and its binding proteins in health and disease. Growth Hormone & IGF Research 13: 113–170. 12914749 10.1016/s1096-6374(03)00038-8

[8] Renehan AG, Zwahlen M, Minder C, O'Dwyer ST, Shalet SM, et al (2004) Insulin-like growth factor (IGF)-I, IGF binding protein-3, and cancer risk: systematic review and meta-regression analysis. The Lancet 363: 1346–1353. 10.1016/S0140-6736(04)16044-3 15110491

[9] Hankinson SE, Willett WC, Colditz GA, Hunter DJ, Michaud DS, et al (1998) Circulating concentrations of insulin-like growth factor I and risk of breast cancer. The Lancet 351: 1393–1396. 10.1016/S0140-6736(97)10384-1 9593409

[10] Pollak M (2000) Insulin-like growth factor physiology and cancer risk. European Journal of Cancer 36: 1224–1228. 10882860 10.1016/s0959-8049(00)00102-7

[11] Benini S, Zuntini M, Manara MC, Cohen P, Nicoletti G, et al (2006) Insulin-like growth factor binding protein 3 as an anticancer molecule in Ewing's sarcoma. International Journal of Cancer 119: 1039–1046. 16570284 10.1002/ijc.21929

[12] Scotlandi K (2006) Targeted therapies in Ewing's sarcoma. Advances in Experimental Medicine and Biology 587: 13–22. 17163152 10.1007/978-1-4020-5133-3_2

[13] Manara MC, Landuzzi L, Nanni P, Nicoletti G, Zambelli D, et al (2007) Preclinical in vivo study of new insulin-like growth factor-I receptor–specific inhibitor in Ewing's sarcoma Clinical Cancer Research. 13: 1322–1330. 10.1158/1078-0432.CCR-06-1518 17317844

[14] Samani AA, Yakar S, LeRoith D, Brodt P (2007) The role of the IGF system in cancer growth and metastasis: Overview and recent Insights Endocrine Reviews. 28: 20–47. 10.1210/er.2006-0001 16931767

[15] Baserga R, Peruzzi F, Reiss K (2003) The IGF-1 receptor in cancer biology. International Journal of Cancer 107: 873–877. 14601044 10.1002/ijc.11487

[16] Olmos D, Postel-Vinay S, Molife LR, Okuno SH, Schuetze SM, et al (2010) Safety, pharmacokinetics, and preliminary activity of the anti-IGF-1R antibody figitumumab (CP-751,871) in patients with sarcoma and Ewing's sarcoma: a phase 1 expansion cohort study The Lancet Oncology. 11: 129–135. 10.1016/S1470-2045(09)70354-7 PMC2941877 20036194

[17] Allison M (2012) Clinical setbacks reduce IGF-1 inhibitors to cocktail mixers. Nature Biotechnology 30: 906–907. 10.1038/nbt1012-906c 23051797

[18] Zhang L, Gardiner BS, Smith DW, Pivonka P, Grodzinsky AJ (2009) Integrated model of IGF-I mediated biosynthesis in deforming articular cartilage. Journal of Engineering Mechanics 135: 439–449.

[19] Zhang L, Gardiner BS, Smith DW, Pivonka P, Grodzinsky AJ (2008) A fully coupled poroelastic reactive-transport model of cartilage. Molecular & Cellular Biomechanics 5: 133–153. 18589501

[20] Yu H, Rohan T (2000) Role of the Insulin-Like Growth Factor Family in Cancer Development and Progression. Journal of the National Cancer Institute 92: 1472–1489. 10995803 10.1093/jnci/92.18.1472

[21] Wilson EM, Rotwein P (2006) Control of MyoD Function during Initiation of Muscle Differentiation by an Autocrine Signaling Pathway Activated by Insulin-like Growth Factor-II. The Journal of Biological Chemistry 281: 29962–29971. 16901893 10.1074/jbc.M605445200

[22] Barta E, Maroudas A (2006) A theoretical study of the distribution of insulin-like growth factor in human articular cartilage. Journal of Theoretical Biology 241: 628–638. 16494900 10.1016/j.jtbi.2006.01.004

[23] Wang HS, Chard T (1999) IGFs and IGF-binding proteins in the regulation of human ovarian and endometrial function. Journal of Endocrinology 161: 1–13. 10194523 10.1677/joe.0.1610001

[24] Chu CH, Tzang BS, Chen LM, Liu CJ, Tsai FJ, et al (2009) Activation of Insulin-Like Growth Factor II receptor induces mitochondrial-dependent apoptosis through G-alpha-q and downstream calcineurin signaling in myocardial cells. Endocrinology 150: 2723–2731. 19095737 10.1210/en.2008-0975

[25] LeRoith D, Roberts CT (2003) The insulin-like growth factor system and cancer Cancer Letters. 195: 127–137. 10.1016/s0304-3835(03)00159-9 12767520

[26] Tavera C, Abribat T, Reboul P, Dore S, Brazeau P, et al (1996) IGF and IGF-binding protein system in the synovial fluid of osteoarthritic and rheumatoid arthritic patients. Osteoarthritis and Cartilage 4: 263–274. 11048623 10.1016/s1063-4584(05)80104-9

[27] Lambert S, Gol-Winkler R, Collette J, Gillis J, Franchimont P, et al (2006) Tumor IGF-II content in a patient with a colon adenocarcinoma correlates with abnormal expression of the gene. International Journal of Cancer 48: 826–830. 10.1002/ijc.2910480607 1713573

[28] Bach LA, Headey SJ, Norton RS (2005) IGF-binding proteins – the pieces are falling into place. Trends in Endocrinology & Metabolism 16: 228–234. 15935690 10.1016/j.tem.2005.05.005

[29] Paye JMD, Forsten-Williams K (2006) Regulation of insulin-like growth factor-I (IGF-I) delivery by IGF binding proteins and receptors. Annals of Biomedical Engineering 34: 618–632. 16547609 10.1007/s10439-005-9064-6

[30] Rajaram S, Baylink DJ, Mohan S (1997) Insulin-Like Growth Factor-Binding Proteins in Serum and Other Biological Fluids: Regulation and Functions. Endocrine Reviews 18: 801–831. 9408744 10.1210/edrv.18.6.0321

[31] Oh Y, Nagalla SR, Yamanaka Y, Kim HS, Wilson E, et al (1996) Synthesis and characterization of insulin-like growth factorbinding protein (IGFBP)-7. Recombinant human mac25 protein specifically binds IGF-1 and -II. Journal of Biological Chemistry 271: 30322–30325. 8939990 10.1074/jbc.271.48.30322

[32] Jones JI, Clemmons DR (1995) Insulin-like Growth Factors and their binding proteins: biological actions. Endocrine Reviews 16: 3–34. 7758431 10.1210/edrv-16-1-3

[33] Zhang L, Gardiner BS, Smith DW, Pivonka P, Grodzinsky AJ (2010) On the role of diffusible binding partners in modulating the transport and concentration of proteins in tissues. Journal of Theoretical Biology 263: 20–29. 20005880 10.1016/j.jtbi.2009.11.023

[34] Bunn RC, Fowlkes JL (2003) Insulin-like growth factor binding protein proteolysis Trends in Endocrinology & Metabolism. 14: 176–181. 10.1016/s1043-2760(03)00049-3 12714278

[35] Busby WH, Nam TJ, Moralez A, Smith C, Jennings M, et al (2000) The complement component C1s is the protease that accounts for cleavage of insulin-like growth factor-binding protein-5 in fibroblast medium. Journal of Biological Chemistry 275: 37638. 10982804 10.1074/jbc.M006107200

[36] Loechel F, Fox JW, G GM, Albrechtsen R, Wewer UM (2000) ADAM 12-S cleaves IGFBP-3 and IGFBP-5 and is inhibited by TIMP-3. Biochemical and Biophysical Research Communications 278: 511–515. 11095942 10.1006/bbrc.2000.3835

[37] Nagase H, Visse R, Murphy G (2006) Structure and function of matrix metalloproteinases and TIMPs. Cardiovascular Research 60: 562–573. 10.1016/j.cardiores.2005.12.002 16405877

[38] Marinaro JA, Casley DJ, Bach LA (2000) O-glycosylation delays the clearance of human IGF-binding protein-6 from the circulation. European Journal of Endocrinology 142: 512–516. 10802531 10.1530/eje.0.1420512

[39] Grimberg A, Cohen P (2000) Role of insulin-like growth factors and their binding proteins in growth control and carcinogenesis. Journal of Cellular Physiology 183: 1–9. 10699960 10.1002/(SICI)1097-4652(200004)183:1<1::AID-JCP1>3.0.CO;2-J PMC4144680

[40] Heding A, Gill R, Ogawa Y, Meyts PD, Shymko RM (1996) Biosensor Measurement of the Binding of Insulin-like Growth Factors I and II and Their Analogues to the Insulin-like Growth Factor-binding Protein-3. The Journal of Biological Chemistry 271: 13948–13952. 8662901 10.1074/jbc.271.24.13948

[41] Headey SJ, Leeding KS, Norton RS, Bach LA (2004) Contributions of the N- and C-terminal domains of IGF binding protein-6 to IGF binding. Journal of Molecular Endocrinology 33: 377–386. 15525596 10.1677/jme.1.01547

[42] Vorwerk P, Hohmann B, Oh Y, Rosenfeld RG, Shymko RM (2002) Binding Properties of Insulin-Like Growth Factor Binding Protein-3 (IGFBP-3), IGFBP-3 N- and C-Terminal Fragments, and Structurally Related Proteins mac25 and Connective Tissue Growth Factor Measured Using a Biosensor. Endocrinology 143: 1677–1685. 11956149 10.1210/endo.143.5.8760

[43] Zhang L, Gardiner BS, Smith DW, Pivonka P, Grodzinsky AJ (2008) IGF uptake with competitive binding in articular cartilage. Journal of Biological Systems 16: 175–195.

[44] Bhakta NR, Garcia AM, Frank EH, Grodzinsky AJ, Morales TI (2000) The Insulin-like Growth Factors (IGFs) I and II Bind to Articular Cartilage via the IGF-binding Proteins. The Journal of Biological Chemistry 275: 5860–5866. 10681577 10.1074/jbc.275.8.5860

[45] Yamanaka Y, Wilson EM, Rosenfeld RG, Oh Y (1997) Inhibition of insulin receptor activation by insulin-like growth factor binding proteins. The Journal of Biological Chemistry 272: 30729–30734. 9388210 10.1074/jbc.272.49.30729

[46] Martel-Pelletier J, Battista JAD, Lajeunesse D, Pelletier J-P (1998) IGF/IGFBP axis in cartilage and bone in osteoarthritis pathogenesis Inflammation Research. 47: 90–100. 10.1007/s000110050288 9562333

[47] Blakesley VA, Scrimgeour A, Esposito D, Roith DL (1996) Signaling via the insulin-like growth factor-I receptor: Does it differ from insulin receptor signaling? Cytokine & Growth Factor Reviews 7: 153–159. 8899293 10.1016/1359-6101(96)00015-9

[48] Slaaby R, Schäffer L, Lautrup-Larsen I, Andersen AS, Shaw AC, et al (2006) Hybrid receptors formed by Insulin Receptor (IR) and Insulin-like Growth Factor I Receptor (IGF-IR) have low Insulin and high IGF-1 affinity irrespective of the IR splice variant. Journal of Biological Chemistry 281: 25869–25874. 16831875 10.1074/jbc.M605189200

[49] Gardiner BS, Smith DW, Pivonka P, Grodzinsky AJ, Frank EH, et al (2007) Solute transport in cartilage undergoing cyclic deformation. Computer Methods in Biomechanics and Biomedical Engineering 10: 265–278. 17671860 10.1080/10255840701309163

[50] Zhang L, Gardiner BS, Smith DW, Pivonka P, Grodzinsky AJ (2007) The effect of cyclic deformation and solute binding on solute transport in cartilage. Archives of Biochemistry and Biophysics 457: 47–56. 17107655 10.1016/j.abb.2006.10.007

[51] Zhang L, Gardiner BS, Smith DW, Pivonka P, Grodzinsky AJ (2010) The Transport of Insulin-like Growth Factor through Cartilage. In: Vafai K, editor. Porous Media: Applications in Biological Systems and Biotechnology: Taylor & Francis Group.

[52] Gardiner BS, Zhang L, Smith DW, Pivonka P, Grodzinsky AJ (2011) A mathematical model for targeting chemicals to tissues by exploiting complex degradation. Biology Direct 6: 1–16. 21936951 10.1186/1745-6150-6-46 PMC3197567

[53] Zhang L (2011) Solute transport in cyclic deformed heterogeneous articular cartilage. International Journal of Applied Mechanics 3: 1–18.

[54] Eviatar T, Kauffman H, Maroudas A (2003) Synthesis of Insulin-Like Growth Factor Binding Protein 3 In Vitro in Human Articular Cartilage Cultures. Arthritis & Rheumatism 48: 410–417. 12571851 10.1002/art.10761

[55] Lee AS (1984) Modeling dynamic phenomena in molecular and cellular biology. Cambridge University Press.

[56] Stumm S, James JM (1996) Aquatic Chemistry. John Wiley & Sons, Inc.

[57] Fong C-C, Wong M-S, Fong W-F, Yang M (2002) Effect of hydrogel matrix on binding kinetics of protein–protein interactions on sensor surface. Analytica Chimica Acta 456: 201–208.

[58] Schneiderman R, Rosenberg N, Hiss J, Lee P, Liu F, et al (1995) Concentration and size distribution of Insulin-like Growth Factor-I in human normal and osteoarthritic synovial fluid and cartilage. Archives of Biochemistry and Biophysics 324: 173–188. 7503553 10.1006/abbi.1995.9913

[59] Qiu Y, Tarbell JM (2000) Numerical Simulation of Oxygen Mass Transfer in a Compliant Curved Tube Model of a Coronary Artery. Annals of Biomedical Engineering 28: 26–38. 10645785 10.1114/1.251

[60] COMSOL-Multiphysics (2007) MA, USA: COMSOL, Inc.

[61] Boden G, Chen X, Kolaczynski JW, Polansky M (1997) Effects of prolonged hyperinsulinemia on serum leptin in normal human subjects. Journal of Clinical Investigation 100: 1107–1113. 9276727 10.1172/JCI119621 PMC508285

[62] Guler H-P, Zapf J, Schmid C, Froesch ER (1989) Insulin-like growth factors I and II in healthy man. Acta Endocrinologica 121: 753–758. 2558477 10.1530/acta.0.1210753

[63] Flessner MF (1996) Small-solute transport across specific peritoneal tissue surfaces in the rat. Journal of the American Society of Nephrology 7: 225–233. 8785391 10.1681/ASN.V72225

[64] Dowthwaite GP, Bishop JC, Redman SN, Khan IM, Rooney P, et al (2004) The surface of articular cartilage contains a progenitor cell population. Journal of Cell Science 117: 889–897. 14762107 10.1242/jcs.00912

[65] Hayes AJ, MacPherson S, Morrison H, Dowthwaite G, Archer CW (2001) The development of articular cartilage: evidence for an appositional growth mechanism. Anatomy and Embryology 203: 469–479. 11453164 10.1007/s004290100178

[66] Sadicka MD, Intintoli A, Quarmby V, McCoy A, Canova-Davisc E, et al (1999) Kinase receptor activation (KIRA): a rapid and accurate alternative to end-point bioassays. Journal of Pharmaceutical and Biomedical Analysis 19: 883–891. 10698554 10.1016/s0731-7085(98)00144-7

[67] Cai L, Okumu FW, Cleland JL, Beresini M, Hogue D, et al (2002) A slow release formulation of insulin as a treatment for osteoarthritis. Osteoarthritis and Cartilage 10: 692–706. 12202122 10.1053/joca.2002.0813

[68] Bach LA (1999) Insulin-like growth factor binding protein-6 the “forgotten” binding protein? Hormone and Metabolic Research 31: 226–234. 10226806 10.1055/s-2007-978723

[69] Stoltz JF (2006) Mechanobiology: Cartilage and Chondrocyte. Amsterdam, The Netherlands: IOS Press.

[70] Brocklehurst R, Bayliss M, Maroudas A, Coysh H, Freeman M, et al (1984) The composition of normal and osteoarthritic articular cartilage from human knee joints. With special reference to unicompartmental replacement and osteotomy of the knee. Journal of Bone & Joint Surgery 66: 95–106. 6690447

[71] Dore S, Pelletier JP, DiBattista JA, Tardif G, Brazeau P, et al (1994) Human osteoarthritic chondrocytes possess an increased number of insulin-like growth factor 1 binding sites but are unresponsive to its stimulation. Possible role of IGF-1-binding proteins. Arthritis & Rheumatism 37: 253–263. 7510486 10.1002/art.1780370215

[72] Sessions CM, Emler CA, Schalch DS (1987) Interaction of Insulin-Like Growth Factor II with Rat Chondrocytes: Receptor Binding, Internalization, and Degradation. Endocrinology 120: 2108–2016. 2436896 10.1210/endo-120-5-2108

[73] Verschure PJ, Marle J, Joosten LA, Berg WB (1995) Chondrocyte IGF-1 receptor expression and responsiveness to IGF-1 stimulation in mouse articular cartilage during various phases of experimentally induced arthritis. Annals of the Rheumatic Diseases 54: 645–653. 7677441 10.1136/ard.54.8.645 PMC1009962

[74] Osipo C, Sara Dorman AF (2001) Loss of Insulin-like Growth Factor II Receptor Expression Promotes Growth in Cancer by Increasing Intracellular Signaling from both IGF-I and Insulin Receptors. Experimental Cell Research 264: 388–396. 11262195 10.1006/excr.2000.5121

[75] Garcia AM, Szasz N, Trippel SB, Morales TI, Grodzinsky AJ, et al (2003) Transport and binding of insulin-like growth factor I through articular cartilage. Archives of Biochemistry and Biophysics 415: 69–79. 12801514 10.1016/s0003-9861(03)00215-7

[76] Mauck RL, Hung CT, Ateshian GA (2000) Modeling of neutral solute transport in a dynamically loaded porous permeable gel: implications for articular cartilage biosynthesis and tissue engineering. Journal of Biomechanical Engineering 125: 602–614. 10.1115/1.1611512 PMC2854001 14618919

[77] Slaaby R, Schaffer L, Lautrup-Larsen I, Andersen AS, Shaw AC, et al (2006) Hybrid receptors formed by insulin receptor (IR) and insulin-like growth factor I receptor (IGF-IR) have low insulin and high IGF-1 affinity irrespective of the IR splice variant. The Journal of Biological Chemistry 281: 25869–25874. 16831875 10.1074/jbc.M605189200

